# Comparative genomic analysis of retrogene repertoire in two green algae *Volvox carteri* and *Chlamydomonas reinhardtii*

**DOI:** 10.1186/s13062-016-0138-1

**Published:** 2016-08-04

**Authors:** Marcin Jąkalski, Kazutaka Takeshita, Mathieu Deblieck, Kanako O. Koyanagi, Izabela Makałowska, Hidemi Watanabe, Wojciech Makałowski

**Affiliations:** 1 Institute of Bioinformatics, Faculty of Medicine, University of Muenster, 48149 Muenster, Germany; 2 Graduate School of Information Science and Technology, Hokkaido University, Sapporo, 060-0814 Japan; 3 Department of Bioinformatics, Faculty of Biology, Adam Mickiewicz University, 61-614 Poznań, Poland; 4 Present address: Bioproduction Research Institute, National Institute of Advanced Industrial Science and Technology (AIST) Hokkaido, Sapporo, 062-8517 Japan; 5 Present address: Julius Kühn-Institute, Institute for Resistance Research and Stress Tolerance, 06484 Quedlinburg, Germany

**Keywords:** Retroposition, Retrogenes, Green algae, *Volvox*, *Chlamydomonas*, Multicellularity, Comparative genomics

## Abstract

**Background:**

Retroposition, one of the processes of copying the genetic material, is an important RNA-mediated mechanism leading to the emergence of new genes. Because the transcription controlling segments are usually not copied to the new location in this mechanism, the duplicated gene copies (retrocopies) become pseudogenized. However, few can still survive, e.g. by recruiting novel regulatory elements from the region of insertion. Subsequently, these duplicated genes can contribute to the formation of lineage-specific traits and phenotypic diversity. Despite the numerous studies of the functional retrocopies (retrogenes) in animals and plants, very little is known about their presence in green algae, including morphologically diverse species. The current availability of the genomes of both uni- and multicellular algae provides a good opportunity to conduct a genome-wide investigation in order to fill the knowledge gap in retroposition phenomenon in this lineage.

**Results:**

Here we present a comparative genomic analysis of uni- and multicellular algae, *Chlamydomonas reinhardtii* and *Volvox carteri*, respectively, to explore their retrogene complements. By adopting a computational approach, we identified 141 retrogene candidates in total in both genomes, with their fraction being significantly higher in the multicellular *Volvox*. Majority of the retrogene candidates showed signatures of functional constraints, thus indicating their functionality. Detailed analyses of the identified retrogene candidates, their parental genes, and homologs of both, revealed that most of the retrogene candidates were derived from ancient retroposition events in the common ancestor of the two algae and that the parental genes were subsequently lost from the respective lineages, making many retrogenes ‘orphan’.

**Conclusion:**

We revealed that the genomes of the green algae have maintained many possibly functional retrogenes in spite of experiencing various molecular evolutionary events during a long evolutionary time after the retroposition events. Our first report about the retrogene set in the green algae provides a good foundation for any future investigation of the repertoire of retrogenes and facilitates the assessment of the evolutionary impact of retroposition on diverse morphological traits in this lineage.

**Reviewers:**

This article was reviewed by William Martin and Piotr Zielenkiewicz.

**Electronic supplementary material:**

The online version of this article (doi:10.1186/s13062-016-0138-1) contains supplementary material, which is available to authorized users.

## Background

Duplication of genetic material is a primary source of evolutionary novelties like origination of new genes [1–4] and retroposition is one of such mechanisms. In this RNA-based duplication process, mRNA is reverse-transcribed to DNA and inserted into a new genomic location, usually a different chromosome (Additional file 1: Figure S1A; reviewed in [5]). The source gene of the duplicated mRNA is often called a parental gene and the products of RNA-based gene duplication, if functional, are called retrogenes and usually are intronless. However, in most of the cases RNA-based duplicated copies (called retrocopies) are nonfunctional because they lack original regulatory elements such as upstream promoters, and thus most of retrocopies encounter silencing and pseudogenization within a few million years and only a few survive [3]. That is why they are considered as ‘dead-on-arrival’ [5]. These duplicated copies of their parental genes that avoided being swept from the genome, evolve faster under relaxed purifying selection, and can acquire new and useful functions (neofunctionalization) or take over a subfunction of the progenitor gene (subfunctionalization), for example become expressed in one specific tissue [6]. Sometimes, the parental gene might even become pseudogenized and completely lost over time, and be replaced by the retrocopy. Such events turn retrogenes into ‘orphan’, i.e., without their parental genes present in the genome [7]. Because of these diverging fates of retrocopies, they are often called as ‘seeds of evolution’ [8] as they can serve as an important source of species-specific traits.

*Volvox carteri* (hereafter *Volvox*), a multicellular green alga commonly found in freshwater habitats, is a widely used model organism in studying the evolutionary transition from unicellular organisms to the multicellular ones [9, 10]. A single individual consists of thousands of somatic cells forming a sphere, inside which several (~16) germ cells called gonidia reside. Its close unicellular relative, *Chlamydomonas reinhardtii* (hereafter *Chlamydomonas*), is about 10 μm in size, has two anterior flagella, and, over the years, it also served as a model organism in the research areas of chloroplast-based photosynthesis, cilliary structure, functions and diseases [11–13]. The *Chlamydomonas* life cycle consists of two phases - a swimming phase in which the cells grow, and a reproductive phase; in contrast, there are two different types of cells that have taken over the above two functions in *Volvox* [10]. Both algae belong to a group of highly adaptable species called chlorophytes. The time of divergence between *Volvox* and *Chlamydomonas* lineages has been estimated to be ~220 MYA [14], which is the latest date in the divergence between unicellular and multicellular organisms. Most probably the last common ancestor of the volvocine algae existing today was unicellular and resembled the present-day *Chlamydomonas* alga [15].

Both algae had their genomes sequenced and published, namely, *Chlamydomonas* in 2007 [13] and *Volvox* in 2010 [16], and *Chlamydomonas* was the first algal species subjected to a genome project [12]. It is believed that the evolution of multicellularity was mainly driven by the emergence of new protein domains as well as new combinations of already pre-existing domains [17]. However, an initial comparative genomics study used for investigating how multicellularity could have evolved in the green algae, revealed that, on the contrary to most of metazoan lineages, there were no significant differences in the protein domain repertoire between *Volvox* and *Chlamydomonas* [16]. The size of their genomes and the number of encoded genes were similar to each other (138 Mbp, 14,520 protein-coding genes, and 118 Mbp, 14,516 protein-coding loci in *Volvox* [16] and *Chlamydomonas* [13], respectively). Therefore, it was speculated that multicellular *Volvox* might possess some minor modifications of lineage-specific proteins that attribute to its increased organismal complexity and different lifestyle. Prochnik et al. [16] concluded that the expansion of lineage-specific proteins composing extracellular matrix and involved in the cell cycle could probably explain the observed morphological differences between these two model organisms.

The genome annotations of the algae have been continuously updated since the initial releases used for the comparative genomic study by Prochnik et al. [16]. In those annotations of the studied algae, the fraction of intronless genes in the genomes differed slightly. There were 8 % of genes without introns in *Volvox*, and 9 % in *Chlamydomonas.* However, the later releases of both algal genome annotations verified the previously estimated numbers. At the time of the presented here study, there were 14,971 protein-coding loci in *Volvox* and 17,728 in *Chlamydomonas*, and the number of identified intronless genes amounted to 2,305 (15.4 %) in *Volvox* and 1,004 (5.7 %) in *Chlamydomonas* (Table 1; see also Methods). Some of these intronless genes might potentially derive from RNA-based gene duplication events, and thus retropositions might have played an important role in the evolution the volvocine algae.

**Table 1:** Overall representation of the data used for the analysis

Number of exons/CDEs	Number of *Volvox* genes^a^	Number of *Chlamydomonas* genes^a^	Number of *Chlorella* genes^a^
1	2305 (15.40 %)/2397 (16.01 %)	1004 (5.66 %)/1311 (7.40 %)	233 (2.38 %)/240 (2.45 %)
2	1332 (8.90 %)/1289 (8.61 %)	1382 (7.80 %)/1476 (8.33 %)	579 (5.91 %)/577 (5.89 %)
3	1155 (7.71 %)/1146 (7.65 %)	1457 (8.22 %)/1427 (8.05 %)	864 (8.82 %)/874 8.93 %)
4	1196 (7.99 %)/1202 (8.03 %)	1440 (8.12 %)/1462 (8.25 %)	1085 (11.08 %)/1094 (11.17 %)
≥5	8983 (60.00 %)/8937 (59.70 %)	12445 (70.20 %)/12052 (67.98 %)	7030 (71.80 %)/7006 (71.56 %)
Total genes	14,971	17,728	9791
Introns per gene	6.27	8.49	6.09

^a^Number and fraction of genes with N number of exons in their structure, including those with UTR exons (on the left) and number and fraction of genes consisting of N number of coding exons only (on the right), relative to the total number of annotated genes are shown

The availability of whole-genome sequences has made a large-scale analysis of retrogenes possible. Retrogenes have been broadly studied among many species, e.g. in human [7, 18–20], fruit fly [21, 22] or other animals [23–25], and comprehensive resources of the animal retrocopies have been made available recently [18]. There have also been a few cases of such studies in plants, e.g. thale cress [26], poplar [27], and rice [28, 29]. However, currently very little is known about the retrogene landscape in green algae. In the present study, we performed a comparative analysis of *Volvox* and *Chlamydomonas* genomes, with a genome of *Chlorella variabilis* NC64A (hereafter *Chlorella*, [30]) used as outgroup, which was the closest publicly available genome to the studied species, for comprehensive exploration the retrogene complements in the green algae. Here we report 141 retrogene candidates identified in *Volvox* and *Chlamydomonas* and the first attempt to estimate the evolutionary history of their origination in the green algae.

## Results and discussion

### Identification of retrogene candidates in Volvox and Chlamydomonas

Fourteen thousand nine hundred seventy-one *Volvox* and 17,728 *Chlamydomonas* protein sequences, as well as 9,791 *Chlorella* proteins, were used to identify retrogene candidates in *Volvox* and *Chlamydomonas* genomes (Table 1) and the percentage of intronless genes in the *Volvox* genome was much larger than that of *Chlamydomonas* (*P* < 2.2E-16, *χ*^2^ test). The identification schema used in this study is summarized in Additional file 1: Figure S2. We started our process of identifying retrogenes in the two algal genomes by utilizing a simple approach of sequence similarity searches using BLAST software [31] (see Methods for details). Proteomes of *Volvox*, *Chlamydomonas,* and *Chlorella* were scanned using 3,708 amino acid query sequences encoded by single-coding-exon genes (1-CDE genes) derived from *Volvox* and *Chlamydomonas* (Table 1). Taking into account a large evolutionary distance separating the studied species (~220 MYA), BLAST searches were performed using protein sequences, since they saturate less rapidly than nucleotide sequences [32]. Moreover, insertion of a retrocopy to a new genomic locus can be accompanied or followed by a gain of new exon(s) in the upstream region, which in turn can assure its functionality by, e.g., providing new upstream regulatory elements. For that reason, we took into account possible exon/intron gains in the untranslated regions.

With our retrogene identification strategy, we predicted 81 and 60 retrogene candidates in *Volvox* and *Chlamydomonas*, respectively (Table 2 and Additional file 2). Retrogene content in *Volvox* genome was significantly higher than in *Chlamydomonas* (*P* = 6.9 × 10^−3^, *χ*^2^ test). Relationships with the parental genes of the 141 retrogene candidates are summarized in Table 3. We conducted a search for hallmarks of the past retroposition, including a poly-(A) tail and target site duplications (TSDs) at the level of DNA sequence. It resulted in finding 22 retrogene candidates (19 in *Volvox* and 3 in *Chlamydomonas*) with a residual poly-(A) tail, however TSDs were not identified in any of the predicted retrogene candidates. Thus, in most of the cases, only the most explicit indicator of the past retroposition, i.e., the loss of introns, was present. Since poly-(A) tail and TSDs decay over time, these features can usually only be identified in very recent retrocopies [5]. The apparent lack of these additional hallmarks of retroposition in majority of the retrogene candidates identified here indicates that they are most likely not of recent origin.

**Table 2:** The number of retrogene candidates identified in this study

	‘Intact’ retrogenes	‘Incomplete’ retrogenes	Total
*Volvox*	76	5	81
*Chlamydomonas*	55	5	60
Total	131	10	141

**Table 3:** Relationship between the identified retrogene candidates and the source species of their parental genes

Parental gene found in	# of *Volvox* retrogenes	# of *Chlamydomonas* retrogenes
*Volvox*	7	1
*Chlamydomonas*	10	1
*Chlorella*	62	56
*Volvox, Chlamydomonas, Chlorella*	2	2
Total	81	60

**Fig. 1:**
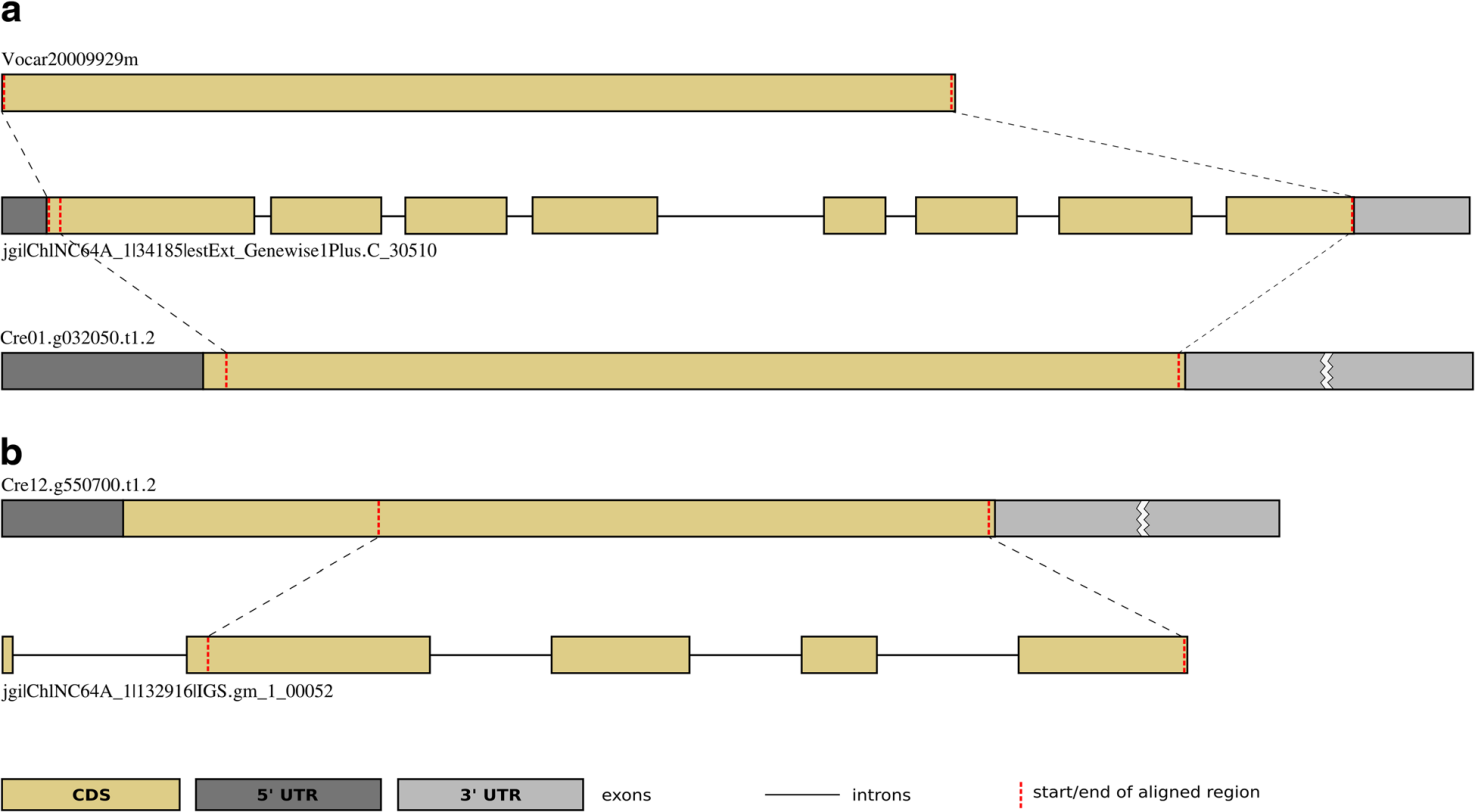
Gene structure comparison between retrogene candidates and their parental genes. **a** Comparison of gene structure between ‘intact’ retrogenes and their parental gene. Two orthologous ‘intact’ retrogene candidates from *Volvox* (*top*) and *Chlamydomonas* (*bottom*) are products of RNA-based duplication of *UDP-galactose transporter* gene. The retroposed region covers all eight protein-coding exons of the parental gene from *Chlorella* (*in the middle*); **b** Example of ‘incomplete’ retrogene as compared to its parental gene. The retroposed region of the *formyl transferase* gene covers only four of the five protein-coding exons of the parental gene found in *Chlorella* (*bottom*). Coding region of the *Chlamydomonas* retrogene (*top*) is longer than that of its parental gene, which likely emerged from the region of insertion

Most of the predicted retrogene candidates were ‘intact’, i.e., comprising of a retroposed region spanning all the introns of their parental genes (Table 2, Fig. 1a and Additional file 1: Figure S1A). However, it has been known that the insertion of retrocopy starts from its 3′ end and it is not always complete - in such a case a part of the 5′ end is lost in the retrocopy (Fig. 1b and Additional file 1: Figure S1B) [5, 22, 33]. In our retrogene identification strategy, we allowed for a certain level of truncation at the 5′ part, including partial loss of parents’ exon/exon boundaries. Such cases were categorized as ‘incomplete’ retrogenes in this study. We identified ten instances of such retrogenes that probably arose from partial (incomplete) retroposition events (Table 2 and Additional file 2). All of them have a complete single exon missing at the 5′ end (N-terminal of the encoded proteins) compared to the exon/intron structure of their parental gene. In four of these cases (two in *Volvox* and two in *Chlamydomonas*), an amino acid sequence produced by the identified ‘incomplete’ retrogene is longer than that of its predicted parent (an example shown in Fig. 1b and Additional file 1: Figure S1C). A gain of new genomic fragment at the 5′ end probably allowed the retrocopy to become functional after the retroposition event. One of the molecular mechanisms that can lead to such N-terminal extension of retrogene is point mutations occurring around the insertion site introducing new upstream start codon. However, we could not find any evidence of such point mutations in the four cases. New genome sequences of the closer relatives of each alga would provide a chance for a detailed analysis of this phenomenon.

Another molecular mechanism that potentially provides retrocopies with functionality is fusion with a fragment of another protein-coding gene. One of the best known cases of such ‘chimeric gene’ is *jingwei*, which has formed from a retroposed copy of alcohol dehydrogenase (*adh*) that merged with several upstream exons of a duplicate of *yellow emperor* gene called *yande* (*ynd*) in the *Drosophila* species [4]. In this study, although we identified no chimeric retrogenes fused with protein-coding regions of other genes, we found ten potentially chimeric retrogene candidates, which probably acquired new exons in their UTRs (either 5′ or 3′). The new UTR exons of retrogenes are not homologous to UTRs of their progenitors nor to any of their nearby upstream/downstream sequences, which suggests that they might have been obtained from the region of retrocopy insertion. Such cases of retrogenes gaining new introns and/or exons were already identified previously e.g., in poplar [27], fruit fly [22] or mammals [34, 35]. The number of similar findings might potentially increase further, since UTRs, especially in the *Volvox* genome, appear to be still not fully annotated. Although we could not find any fusion of protein-coding parts of a retrogene and another gene, this is mainly because our retrogene screening strategy, in which only 1-CDE genes were initial candidates for retrogenes, is not suited for detecting chimeric retrogenes in the first place. The use of a specialized strategy for detecting chimeric retrogenes, like e.g., applied in [28], would be useful and reveal the complement of them in the algae.

### Evolutionary origin of algal retrogenes

To analyze the evolutionary history of the predicted 141 retrogene candidates, we performed phylogenetic analyses of gene families that included the retrogene candidates. Among the set of homologous gene groups of the three algae, the identified retrogene candidates were found to be present in 82 of such groups (for detailed composition see Additional file 1: Table S1). Out of these, 40 included less than four gene sequences and therefore only the remaining 42 groups were subjected to phylogenetic analysis based on the maximum-likelihood approach. The phylogenetic relationships for these gene clusters are depicted in Additional file 1: Figure S3.

**Fig. 2:**
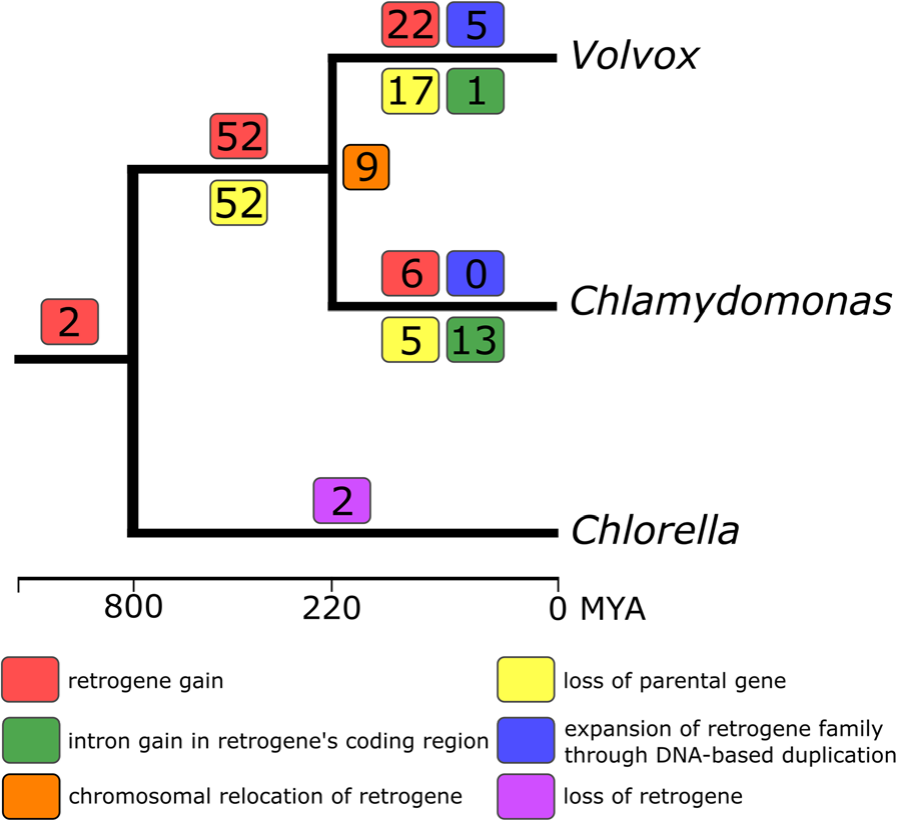
The inferred evolutionary events leading to the emergence of the identified retrogene candidates. The numbers in boxes that are projected on the phylogeny of the three studied green algae represent the estimated count of evolutionary events based on the composition of the retrogene-containing gene clusters, the inferred phylogenetic trees of the retrogene-containing homologous gene families, and the analyzed synteny. Presented divergence dates according to Herron et al. [14]

Based on the composition of the retrogene-containing gene clusters, the inferred phylogenetic trees of the retrogene families, and the analyzed synteny, we reconstructed the history of evolutionary events, such as retroposition, gene duplication and/or loss, in the phylogeny of the studied green algae. In this process, we applied the basic principle of parsimony to effectively minimize the number of the necessary evolutionary steps. If two orthologous retrogene candidates from *Volvox* and *Chlamydomonas* were found, both lying in a syntenic region and having their shared parental gene predicted in the selected outgroup, we assumed that the retroposition event predated the speciation of *Volvox* and *Chlamydomonas*, and was followed by a loss of parental gene in the common ancestor of both algae. Forty-three of such retroposition events in the common ancestor of the two algae were identified, giving birth to ninety-three retrogene candidates (Fig. 2). In case of two retrogene families of this type, subsequent retrogene duplication events took place. Similar to the above, when orthologous retrogenes were found to share the same parental gene, but not to be present in a syntenic region, we assumed that one of the retrogenes had undergone relocation after retroposition. Nine events of this type were found here, giving birth to 18 retrogene candidates (Fig. 2 and Additional file 1: Figure S1D). We assumed that relocation of a retrogene, however difficult to distinguish, is more parsimonious than two independent retroposition events followed by a loss of parental gene in each lineage, as described in a previous study of fruit fly retrogenes [22]. If the parental gene and its resultant retrogene were found to be present in the same species, we assumed an independent, lineage-specific retroposition. In the *Volvox* lineage, there were more of such gains of new retrogenes but, additionally, accompanied by a higher rate of parental gene loss compared to *Chlamydomonas*. We considered that such a loss of parental gene took place, if a retrogene was found in e.g., *Volvox* while its parental gene (multi-exon homolog) was identified only in *Chlamydomonas* with no gene synteny. Such a scenario assuming *Volvox*-specific retroposition followed by a loss of the parental gene in the same lineage is more parsimonious than considering retroposition event in the last common ancestor of the two algae followed by retrogene loss in *Chlamydomonas* and parental gene loss in *Volvox*. In the unicellular *Chlamydomonas*, six lineage-specific retrogene gains were detected. No new retrogenes were further propagated in this lineage by means of DNA-based duplications. In the multicellular *Volvox*, 22 lineage-specific retroposition events took place, with two subsequent duplications that resulted in expansion of the retrogene family (Fig. 2).

Moreover, we found fourteen cases where, most likely, the orthologous retrogene in another alga underwent a subsequent intron gain (Additional file 1: Figure S1E), acquiring either one or two new introns, and thus not identified initially as retrogene candidate by our retrogene-screening strategy. A summary of the above retrogene candidates in present Additional file 1: Table S2. The phenomenon of intron gain events in retrogenes has been already described, e.g., in the poplar genome [27]. Thirteen of such events were discovered in *Chlamydomonas*, and probably in four of them, gain of intron was accompanied by relocation of the orthologous retrogene. Only one example of this phenomenon was discovered in *Volvox* (Vocar20006328m.g), where the orthologous retrogene that acquired an intron was found to lie in a syntenic region to the one of *Chlamydomonas*. Comparison of genomes of the studied green algae (see Methods) shows that there are 6.27 and 8.49 introns per gene in *Volvox* and *Chlamydomonas*, respectively (Table 1). Considering this, we can speculate that gain of introns among the orthologous retrogenes in the *Chlamydomonas* lineage could be the reason behind the above-described results.

### Orphan retrogenes

As recently highlighted by [7], some retrogenes can become ‘orphan’ due to loss or pseduogenization of their parental genes. A general strategy that is usually adopted for identifying retrogenes is to look for pairs of genes with high sequence similarity coming from the same species (genome), where one of them has multiple exons (parental gene) and the other is a single-exon gene (retrogene). In case of ‘orphan’ retrogenes, applying the above strategy will simply fail to identify them. Thus, the only way to find these ‘orphan’ retrogenes is to look for their multi-exon progenitors in other closely related species. In our study we looked for retrogene candidates and their parental genes not only by intra-species searches in *Volvox* and *Chlamydomonas* but also by conducting inter-species searches including data from the three green algae, thus our analyses allowed us to detect the retrogene complement including the ‘orphan’ retrogenes.

Surprisingly, out of the 141 predicted retrogene candidates, 129 had their parental gene missing in the same genome, making them ‘orphan’. This means that most of the identified retrogene candidates could not be detected only by intra-species searches in each genome. In 118 cases, the source gene had been identified in the outgroup species *Chlorella*, which suggests that predicted retrogenes possibly replaced their progenitors. Ten ‘orphan’ retrogene candidates from *Volvox* had their parental gene predicted in the unicellular *Chlamydomonas*. These are probably products of *Volvox* lineage-specific retropositions followed by loss of the source gene, for example by mean of pseudogenization. None of the predicted parental genes from *Chlamydomonas* lies in a syntenic region to a *Volvox* retrogene, implying that these are not examples of orthologous retrogenes that underwent intron gain events. One similar case has been identified for *Chlamydomonas*.

**Fig. 3:**
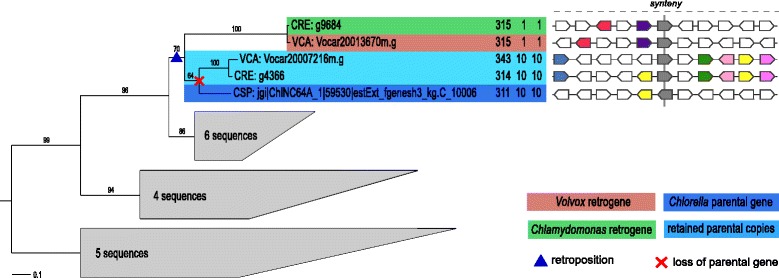
An example of homologous retrogene candidates with their parental genes retained after retroposition. Maximum likelihood tree of the homologous gene group including retrogenes encoding *serine/threonine protein phosphatase PP2A, metallophosphoesterase* is shown on left. Inferred evolutionary events are projected on the phylogeny. Retroposition most likely occurred before speciation of *Volvox/Chlamydomonas* lineage from the outgroup *Chlorella* lineage and was followed by retention of the parental gene in each lineage, while *Chlorella* lost the retrocopy. VCA – *Volvox*; CRE – *Chlamydomonas*; CSP – *Chlorella*. Values in each OTU indicate length of encoded protein (aa), number of exons, and number of coding exons. Bootstrap support values (≥60 %) for the calculated tree are shown on branches. Clades of distant homologs of the retro- and parental genes were collapsed and the number of collapsed sequences is shown within each clade. The tree was rooted using midpoint-rooting approach. Synteny between neighboring genes (five upstream, five downstream) is shown on the right with each color representing homologous genes. Both predicted retrogenes are syntenic to each other

Interestingly, only four retrogene candidates belonging to two groups of orthologous retrogene candidates from *Volvox* and *Chlamydomonas* had their parental genes identified in all three species. Most likely, the parental gene has been retained after retroposition and subsequently passed to both studied algae after the speciation of their lineages (Additional file 1: Figure S1E). One of the described pair of retrogene candidates belongs to the family of serine/threonine protein metallophosphoesterases and its phylogenetic tree is depicted in Fig. 3. In general, one copy of a duplicated gene pair will be under relaxed selection and, by accumulating mutations, it can become nonfunctional - pseudogenized [3, 36, 37]. However, maintaining a second copy of a gene, although rarer, can be beneficial to the species [37, 38]. In all other cases, orphaning of the resultant retrogene candidates took place, which might suggest their beneficial role to both algal genomes and thus the observable displacement of the parental genes [39].

### Functional annotation of the identified retrogenes

Apart from identification and phylogenetic analysis of retrogene candidates, we also examined their Gene Ontology (GO) categories as well as those of their parental genes for finding if any functional differences exist between them. The range of the annotated functions is versatile, e.g., protein binding, structural constituent of ribosome, hydrolase activity or electron transporter activity. Regrettably, the function of many retrogene candidates and parental genes remains unknown (Additional file 3). Among the genes for which the function is annotated, we could observe that retrogene candidates share the same functional categories as their parental genes. Consequently, we did not observe any cases where the annotated function of retrogene candidate differed from the one of its progenitor. This does not allow us to assume that any examples of neofunctionalization in retrogenes had occurred. One of the reasons is that these functional annotations are based on sequence similarity only. For further investigating neofunctionalization and subfunctionalization in the retrogene candidates, performing additional experiments would be useful, e.g., comparison of gene expression patterns between the parental gene and the retrogene by using RNA-seq could highlight the specialized retrogene expression in a different cell type or developmental period.

In addition to functional annotation, we further assessed the functionality of identified retrogene candidates by detecting functional constraint on the identified retrogene candidates measured with the ratio of the number of nonsynonymous substitutions per nonsynonymous site (*d*_*N*_) versus the number of synonymous substitutions per synonymous site (*d*_*S*_), *d*_*N*_*/d*_*S*_. Most of gene retrocopies are ‘dead-on-arrival’ and only a few become functional [5]. Therefore under an assumption that a retrocopy is functionless while its parental gene is functional, it is expected that the *d*_*N*_*/d*_*S*_ ratio should be equal to or higher than 0.5 [21]. We calculated the *d*_*N*_*/d*_*S*_ ratios for all pairs of retrogene candidates and their parental genes predicted here and examined the functionality of retrogene candidates with the stringent criteria, i.e., the *d*_*N*_*/d*_*S*_ <0.5. As a result, we observed that in most of the cases the predicted retrogene candidates showed statistically significant signature of functional constraint, implying that these are probably functional (Additional file 2). These genes didn’t have their parental gene in the same species making them ‘orphan’, which agrees with observations from the previous study in human [7]. We note that for most of pairs of retrogene candidates and their parental genes, very large *d*_*S*_ values were observed (Additional file 2). These values might indicate saturation of synonymous substitutions, thus potentially causing biased estimates of *d*_*N*_*/d*_*S*_. Since the *d*_*S*_ value reflects the time after the divergence of the two sequences in general, these were consistent with our phylogenetic analyses indicating ancient retroposition events in most cases (Fig. 2). A follow-up study with the use of genomes of species that are phylogenetically closer to *Volvox* and *Chlamydomonas* as outgroup would allow us to produce acceptable *d*_*S*_ values and perform a much more reliable assessment of the functionality of retrogenes. The results of this *d*_*N*_*/d*_*S*_ analysis could suggest a possibility of a functional takeover of the parental genes’ function by the retroposed genes, leading to their marginalization and displacement [39].

### Retrogenes and the evolution of multicellularity

**Fig. 4:**
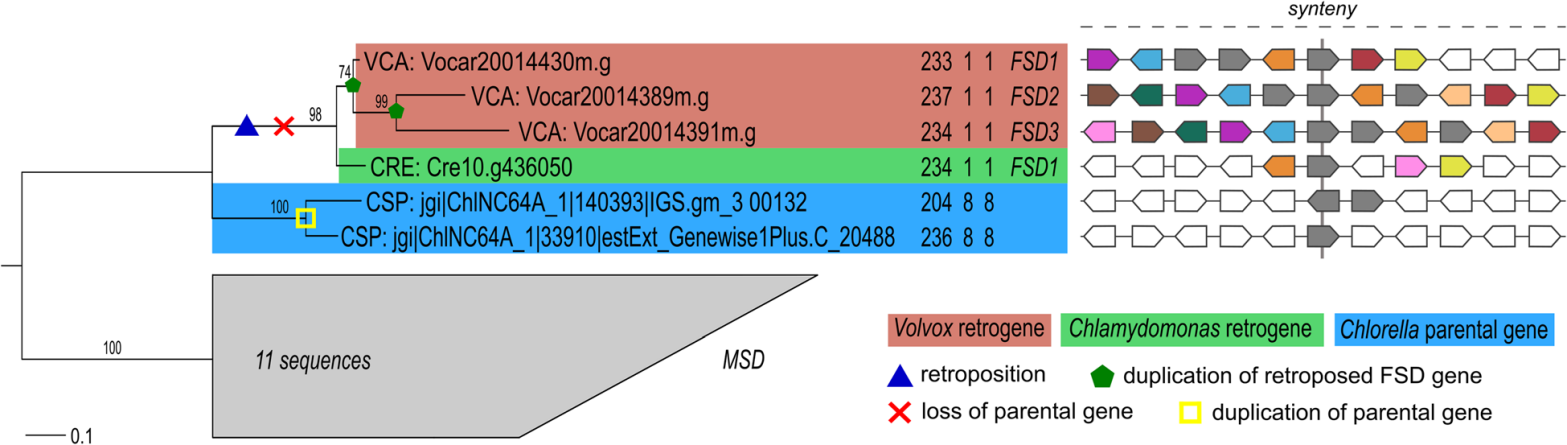
Expansion of the *FSD/MSD* (*iron/manganese superoxide dismutase*) gene family after retroposition. Maximum likelihood tree of the homologous gene group including *FSD*/*MSD* genes is shown on left. *FSD* genes in the *Volvox*/*Chlamydomonas* lineage derived from retroposition followed by loss of parental gene in the common ancestor and experienced two duplication events in *Volvox* lineage. VCA - *Volvox*; CRE - *Chlamydomonas*; CSP - *Chlorella*. Values in each OTU indicate length of encoded protein (aa), number of exons, number of coding exons, and gene name as annotated in the Phytozome database. Bootstrap support values (≥60 %) for the calculated tree are shown on branches. Predicted evolutionary events are projected on the phylogeny. The *MSD* clade is collapsed and the number of collapsed sequences is shown within it. The tree was rooted using midpoint rooting approach. Synteny conservation of neighboring genes (five upstream, five downstream) of the *FSD* genes is shown to the right with each color representing homologous genes

Multicellularity is one of the most important innovations in the evolution of life. For studying the evolution of multicellularity, the volvocine green algae have been considered as an ideal and rare model system because of the evolutionary close relationship with unicellular organisms [9]. In addition to the lineage-specific DNA-based gene duplications [40], RNA-based gene duplications (retropositions) can also serve as important sources of evolutionary novelty, contributing to phenotypic effects of a species by producing genes with modified or completely new functions [5]. In the light of our above-described findings on retrogene repertoire in the two green algae, we investigated whether retropositions could have contributed to the evolution of multicellularity in this lineage. Based on the conducted analyses, it is clear that *Volvox* lineage contains significantly larger number of retrogenes, while having a smaller number of genes encoded by its genome compared to the unicellular *Chlamydomonas* (see “*Identification of retrogene candidates in Volvox and Chlamydomonas*” section), indicating possible contributions of retrogenes to the morphological differences in the two algae. Nevertheless, none of the genes thought to contribute to the observed morphological differences [16] was found among our retrogene set (Additional files 2 and 3). We found only two families of retrogenes that underwent expansion, namely the iron/manganese superoxide dismutase (Fig. 4) and small nuclear ribonucleoprotein SmE family, both of them identified in the *Volvox* lineage. These results suggest that the gene family expansions described by Prochnik et al. [16] were generally independent of retroposition events and that retroposition could be one of the probable molecular mechanisms contributing to the evolution of multicellularity in the green algae.

Both studied algae do not differ significantly in terms of the annotated functions of their predicted retrogene candidates. However, it has been reported previously that two retrogene candidates from *Volvox*, *fer1* (Vocar20002398m.g) and *fsd1* (Vocar20014430m.g), show different expression patterns between somatic cells and germ cells called gonidia [41]. *fer1* is a gene encoding ferredoxin, which belongs to a protein family containing Fe-S clusters and plays an important role in the transport of electrons during photosynthesis [42]. *fsd1* is an iron superoxide dismutase, which is an enzyme playing an important role in antioxidant defense system [43]. It has been confirmed by the real-time RT-PCR analyses that both retrogenes show an explicitly higher expression rate in gonidia as compared with somatic cells [41]. Unfortunately, the expression patterns for the parental genes of *fsd1* and *fer1* could not be assessed, since both retrogene candidates were predicted to be orphan with no parental gene present in the same genome (Additional file 2). However, interestingly, our phylogenetic analysis showed that *fsd1* experienced DNA-based duplication twice in *Volvox* lineage, resulting in two more copies existing in *Volvox*, namely *fsd2* (Vocar20014389m.g) and *fsd3* (Vocar20014391m.g) (Fig. 4). Comparing the expression patterns of all three *Volvox fsd* genes and their single *Chlamydomonas* ortholog (*fsd1*, Cre10.g436050.t1.2) could prove whether these retrogenes contributed to the observed morphological differences of the algae.

The significant difference in the single-exon genes content between genomes of multicellular *Volvox* and unicellular *Chlamydomonas* hinted that some of them might have been potentially derived from RNA-based duplications, and therefore they were analyzed here. Whether retrogenes were truly responsible for any observable differences between phenotypes of *Volvox* and *Chlamydomonas* remains debatable. The same, however, applies to the previously hinted set of protein families that expanded in the *Volvox* lineage. A recent genome-wide analysis has shown that alternative splicing patterns are different between these two green algae, indicating an important role of alternative splicing for expansion of organismal complexity during evolution of multicellularity in the green algae [44]. Interesting and still understudied area is how genes are tweaked and how is their expression regulated in both algae. Changes in the gene control regions that bind transcription factors can affect the time and place at which the encoded proteins are synthetized. Checking the non-protein-coding content might bring some interesting answers to the question of how the organismal complexity arose in the multicellular *Volvox* and might actually be much more suitable for such analyses.

## Conclusions

Despite the current availability of many genomic sequences of green algae such as chlorophytes, our knowledge about algal retrogenes is still very scarce. Here we presented the first attempt to catalogue the retrogene repertoire in green algae, resulting in identification of 81 retrogene candidates in *Volvox* and 60 in *Chlamydomonas* including many ‘orphan’ retrogenes. Almost all of them seem to be functional based on the stringent functionality criteria, which is an important finding since every newly inserted retrogene that was not eliminated from the genome can subsequently contribute to the evolution of a gene with modified or a completely different function from the original one. However, functional information about retrogenes and their parental genes in the green algae is still far from being comprehensive and, therefore, we could not clearly demonstrate the contribution of retroposition to the evolution of multicellularity in this lineage. To resolve this issue, genome-wide gene expression and functional analyses would be necessary. The current results are only first estimate of the evolutionary history of retrogene origination in green algae, yet we believe that presented study will provide a good foundation for any future investigation of the retrogene repertoire in this lineage, especially by applying modified search criteria, e.g., for finding chimeric genes as products of fusion of protein-coding parts of a retrogene.

## Methods

### Genomic dataset of the studied algae

Genomic data, nucleotide and protein sequences, and gene annotations for *V. carteri* and *C. reinhardtii* were obtained from the Phytozome v8.0 [45, 46]. For *Chlamydomonas* we used the data from Augustus update u11.6 annotation of JGI assembly v5.3 with a total of 19,529 protein-coding transcripts (17,728 protein-coding loci, 1,801 products of alternative splicing). For *Volvox,* we used JGI annotation 2.0 on assembly v2 with 14,971 loci containing protein-coding transcripts (15,285 total transcripts, 314 alternatively spliced transcripts). Data for the outgroup species, *C. variabilis* NC64A, were obtained from the Joint Genome Institute database [47] and included 9,791 gene models [30]. The data used in this study are summarized in Table 1. Alternative transcripts were purged from the dataset, leaving only the data of the longest transcript from each protein-coding locus. Gene ontology (GO) terms, transposons annotation and synteny information were also taken from the Phytozome database, and for the synteny check between the outgroup *Chlorella* and the other studied algae we used additional information from the JGI database.

### Identification of retrogene candidates

The scheme for identification of retrogene candidates is summarized in Additional file 1: Figure S2. Amino acid sequences encoded by 1-CDE genes (genes with a single protein-coding exon) from *Volvox* and *Chlamydomonas* (candidate set) were, similarly to other studies [7, 26], initially purged from sequences of histone genes, as their intronless state is related to an ancient gene structure rather than to retroposition. Next, we used them as queries for a BLASTP search [31] against proteomes of all three analyzed algal species (protein subject set) with a cutoff *E*-value at 0.001. The obtained results were mapped to exon-intron structures of the genes and filtered to keep only the pairs where a 1-CDE gene – retrogene candidate matched a multi-exon gene - parental gene. In the additional criterion, we demanded that a potential retrogene candidate and its parental gene had to share at least 50 per cent amino acid identity in the aligned region, which spans over 50 per cent of their length and at least 35 amino acids. Since it is known that the insertion of a retrocopy into a new genomic location starts from the 3′ end of the transcript and sometimes can be incomplete [5], we required that the BLASTP-derived aligned region between a retrogene candidate and its parental gene covered at least two 3′ terminal exon-exon junctions of the parental gene. This allowed us to exclude 1-CDE genes derived, e.g., from adjacent exons merging. We discarded any cases involving possible DNA-based duplications by removing predicted retrogene candidates with 50 per cent of their sequences overlapping with transposable elements (adopted from [27, 48]). Additionally, for the same reason, all retrogenes with their neighboring genes (minimum one) homologous to those of their progenitors were removed to exclude the products of segmental duplications. Conservation of the gene-neighborhood of retrogene and its parental gene was inspected by comparing five upstream and five downstream genes.

Finally, we conducted an additional search for the presence of poly-(A) tail of minimum 8 bps within the 3′ UTRs of retrogene candidates, plus 500 bp downstream region from the 3′ end (adopted from [26]). In cases of lack of 3′ UTR annotation, we took an average length calculated from all annotated 3′ UTRs in a given genome and added 500 bp of a downstream sequence. We also examined retrogenes for a presence of Target Site Duplications (TSDs), another hallmark of retroposition. These are regions of 4–6 bp in length, flanking retroposed sequence upstream and downstream of the 5′ and 3′. For that task we used the LTRharvest software [49].

### Calculation of *d*_*N*_*/d*_*S*_ ratio

To examine the functionality of the identified retrogene candidates, we calculated the ratio of the number of nonsynonymous substitutions per nonsynonymous site (*d*_*N*_) versus the number of synonymous substitutions per synonymous site (*d*_*S*_), *d*_*N*_*/d*_*S*_, with codeml program of the PAML package, version 4.9 [50]. The regions of resultant BLASTP alignment between pairs of a retrogene candidate and its predicted parental gene were used to calculate the ratio after converting each amino acid alignment into a corresponding codon alignment with PAL2NAL [51]. The calculation of *d*_*N*_*/d*_*S*_ was performed twice for each gene pair; first under fixing the ratio to 0.5 and second with estimating the ratio, and the difference of log likelihood values was used for a likelihood ratio test. If more than one parental gene was identified in the same genome for a given retrogene candidate by our retrogene identification strategy, we first calculated *d*_*S*_ in all parent-retrogene pairwise combinations and considered one parental gene with the smallest *d*_*S*_ as the representative parental gene for calculating the *d*_*N*_*/d*_*S*_ ratio.

### Functional annotation of retrogene candidates

For some genes that lacked functional annotation but had their Pfam domain predicted, we used the ‘pfam2go’ mapping [52, 53] to assign GO terms. Additionally, we employed GOanna tool, which is a part of the AgBase resource [54]. To annotate GO terms we set GOanna to screen General GO Databases like UniProt, SwissProt, TrEMBL as well as one of the Custom databases, namely ‘Plant’.

### Phylogenetic analyses

First, we built homologous groups of proteins from all three analyzed species. Such homology groups were constructed using a standalone version of InParanoid [55] which uses BLAST all-against-all sequence comparisons. We ran the program first to construct all possible pairwise comparisons of proteomes and retrieve pairwise homology groups between *Volvox*, *Chlamydomonas*, and *Chlorella.* Subsequently, we applied a single-linkage approach to perform merging of the constructed pairwise clusters in order to obtain multi-species groups of homologous sequences.

Phylogenetic trees of multi-species homologous groups that included the predicted retrogene candidates were built based on alignments of amino acid sequences constructed with MAFFT v6.953b [56] using the *L-INS-i* strategy [57]. We used RAxML v7.2.8 [58] to infer phylogenetic relationships for the constructed groups with at least four members, and including one or more of the predicted retrogene candidates. The software was executed with a rapid bootstrapping algorithm [59] and the amino acid substitution matrices as well as the amino acids’ frequencies were estimated from the input alignments. Because of lack of ancestral branches, in order to construct a balanced rooting of trees, all of them were rooted using midpoint rooting approach (placing the root at the mid-point of the longest distance between two terminal nodes). Visualization of the trees was performed with a Python programming language library called ETE [60]. Additional editing of generated images was done manually using and open source graphics editor Inkscape [61].

## Reviewers’ comments

### Reviewer’s report 1: William Martin. University of Düsseldorf, Germany

#### Reviewer summary

This is a thorough and sound characterization of retrogenes in the *Volvox* lineage. It is a valuable contribution to the literature in that field. It should be published.

Author’s response: *We would like to thank Dr. Martin for reviewing this manuscript and recommending it for publication.*

### Reviewer’s report 2: Piotr Zielenkiewicz. Institute of Biochemistry and Biophysics, PAS, Poland

#### Reviewer summary

Jąkalski et al. conducted a comparative analysis of two algae: *Chlamydomonas reinhardtii* and *Volvox carteri*. The authors identified retrogenes in both genomes, concentrating their efforts on distribution, functional annotations and evolutionary history of retrogenes. Moreover, the authors conducted bioinformatics analysis to confirm functionality of predicted retrogenes, emphasizing their possible impact on diverse morphological traits in analyzed algae. The manuscript is well written and nicely organized.

#### Reviewer recommendations to authors

The authors used estimated evolutionary rate of retrogenes dN/dS ratio, mentioning that high dS values were observed possibly due to high evolutionary distance separating the studied species. Unfortunately, saturation of dS values can substantially bias the dN/dS ratio estimation. How did authors deal with this issue? Did they set the threshold on dS values taken into account, filtering out cases with unreliable values? If not I suggest to reproduce dN/dS analyses with recommendations suggested by Villanueva-Cañas et al. [Villanueva-Cañas JL, Laurie S, Albà MM. Improving genome-wide scans of positive selection by using protein isoforms of similar length. Genome Biol Evol. 2013;5(2):457–67. doi: 10.1093/gbe/evt017. PubMed PMID: 23377868; PubMed Central PMCID: PMC3590775].

Author’s response: *We agree with reviewer’s comment that saturation of d*_*S*_*values can bias the d*_*N*_*/d*_*S*_*estimates and that such cases should be filtered out from the analyses. However, suggested criteria seemed to be too stringent for our retrogenes, most of which lack their parental genes in the same genome. Therefore, for all retrogenes, we presented d*_*S*_*value and d*_*N*_*/d*_*S*_*ratio in the additional file 2, so that readers can easily recognize a potential problem of high d*_*S*_*values. However, we believe that the recommendations suggested by Villanueva-Cañas* et al. *for the similar type of analysis, do not entirely apply in our study. None of the genes from the outgroup species Chlorella has annotations of transcript/protein isoforms. In addition, among the retrogene candidates and parental genes identified in Volvox and Chlamydomonas, we do not find any of them to possess isoforms either. A follow-up study with the use of closer genomes to those of Volvox and Chlamydomonas as an outgroup would allow a more reliable assessment of the functionality of retrogenes. Based on this reviewer’s comment, we have revised the end of the “Functional annotation of the identified retrogenes” section.*

The authors chose *Chlorella variabilis NC64A* as the outgroup in subsequent analyses of *Volvox* and *Chlamydomonas* genomes? What was the rationale to choose *Chlorella* in this case?

Author’s response: *At the time of performing this study, the Chlorella genome was the closest available to those of Volvox and Chlamydomonas, therefore we chose it as an outgroup for our analyses. We have revised the manuscript to include this information.*

Minor issues:

- Page 1, row 37: missing words between ‘higher’ and ‘the’
- Page 5, row 2: “Those authors” seems to be imprecise
- Page 6, row 3: change to Arabidopsis
- Page 6, row 10: add) after]
- Page 17, row 55: remove hyphen between ‘candidate’ and ‘matched’

Author’s response: *Thank you for pointing out these mistakes. We have made corrections as suggested.*

## Abbreviations

1-CDE, single-coding-exon; CDE, coding-exon; *d*_*N*_, the number of nonsynonymous substitutions per nonsynonymous site; *d*_*S*_, the number of synonymous substitutions per synonymous site; GO, Gene Ontology; TSD, target site duplication

## Additional files

Additional file 1:
**Figure S1.** Various scenarios of retroposed genes. Figure S2. Maximum likelihood trees of the multi-species clusters containing retrogene candidates constructed with InParanoid. Figure S3. Identification of retrogene candidates in *Volvox* and *Chlamydomonas* genomes. Table S1. Composition of multi-species homologous genes clusters containing the predicted retrogene candidates, constructed using InParanoid. Table S2. Identified retrogene candidates and their homologs that are most likely retrogenes that underwent intron gain events. (DOCX 5396 kb) Additional file 2: Detailed information on all predicted retrogene candidates. (XLSX 45 kb) Additional file 3: Predicted functions (GO terms) of the retrogene candidates. (XLSX 24 kb)
