# Supplementary material for: Comparative genomic analysis of retrogene repertoire in two green algae Volvox carteri and Chlamydomonas reinhardtii
Source: Biol Direct. 2016 Aug 4;11:35. doi: 10.1186/s13062-016-0138-1 (PMC4972966; doi:10.1186/s13062-016-0138-1)
Supplement: Additional file 1: — Figure S1. Various scenarios of retroposed genes. Figure S2. Maximum likelihood trees of the multi-species clusters containing retrogene candidates constructed with InParanoid. Figure S3. Identification of retrogene candidates in Volvox and Chlamydomonas genomes. Table S1. Composition of multi-species homologous genes clusters containing the predicted retrogene candidates, constructed using InParanoid. Table S2. Identified retrogene candidates and their homologs that are most likely retrogenes that underwent intron gain events. (DOCX 5396 kb) [file 13062_2016_138_MOESM1_ESM.docx]

**
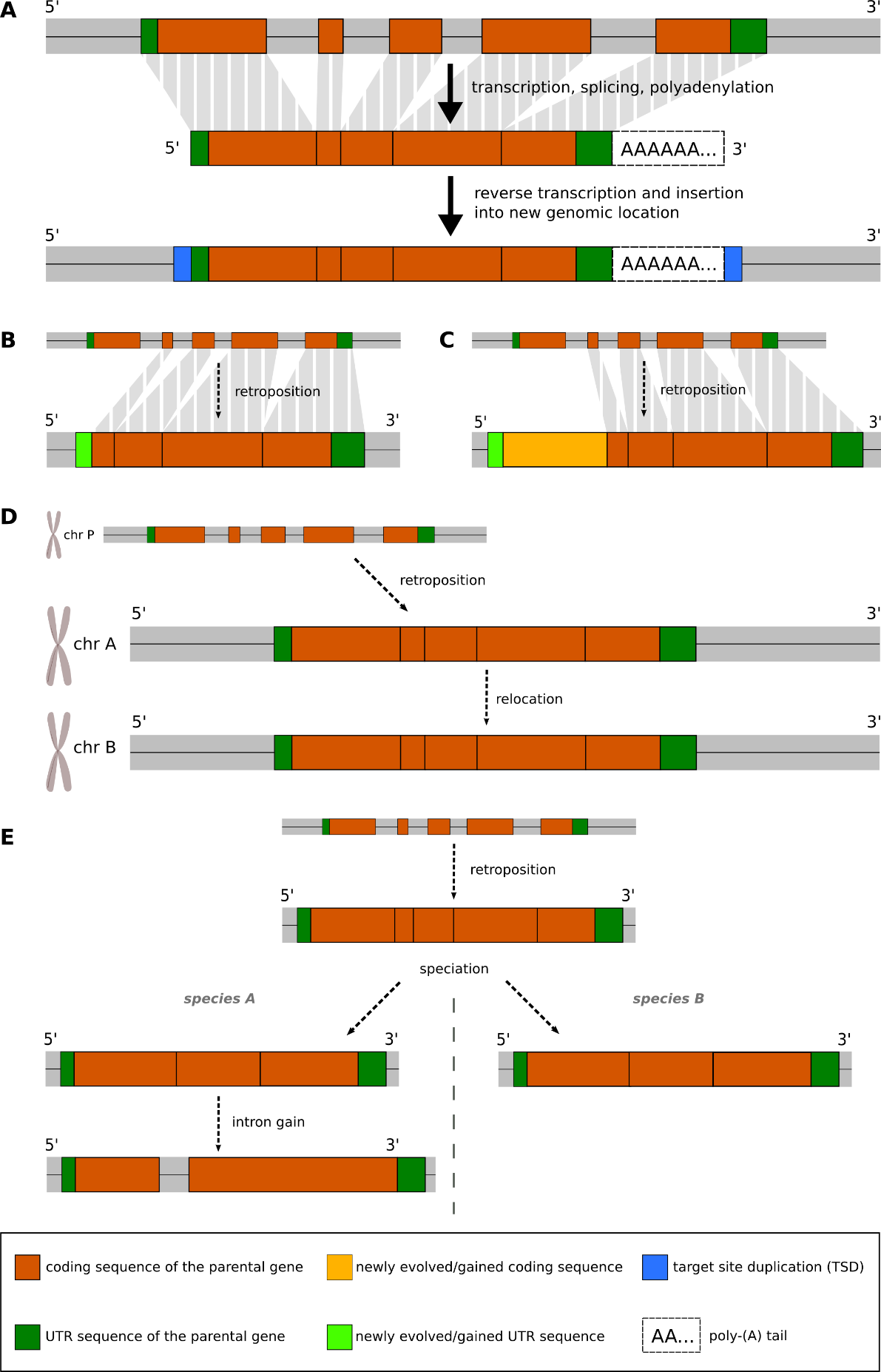
**

**Figure S1. Various scenarios of retroposed genes**. **A.** basic mechanism of retroposition, **B.** incomplete retroposition with truncation at the 5' site, **C.** incomplete retroposition with truncation of parental sequence at the 5' site; gain of new coding sequence and UTR sequence at 5' site, **D.** retroposition followed by relocation of retroposed gene, **E.** retroposition followed by intron gain in retrogene.

**group_37**

**
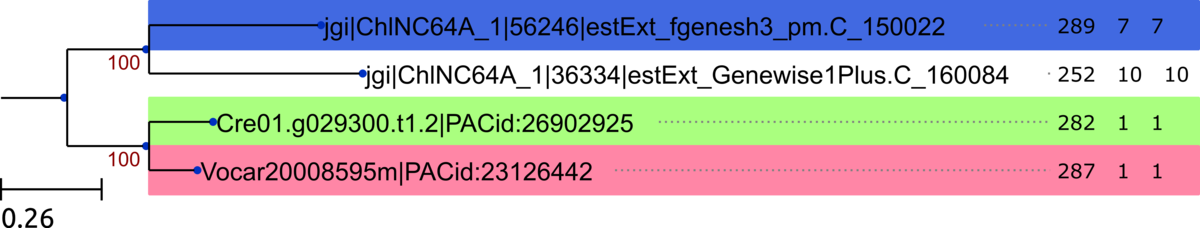
**

**group_38**

**
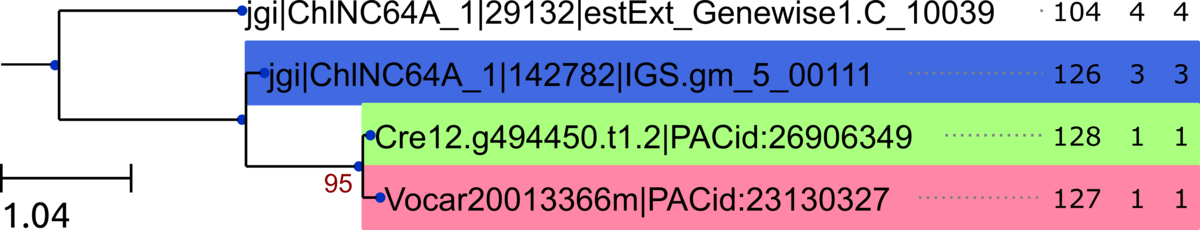
**

**group_39**

**
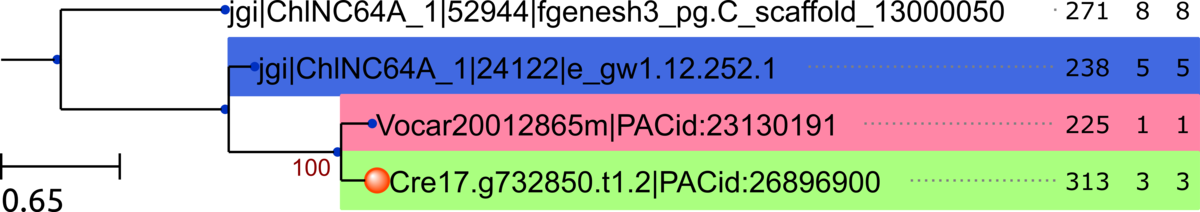
**

**group_40**

**
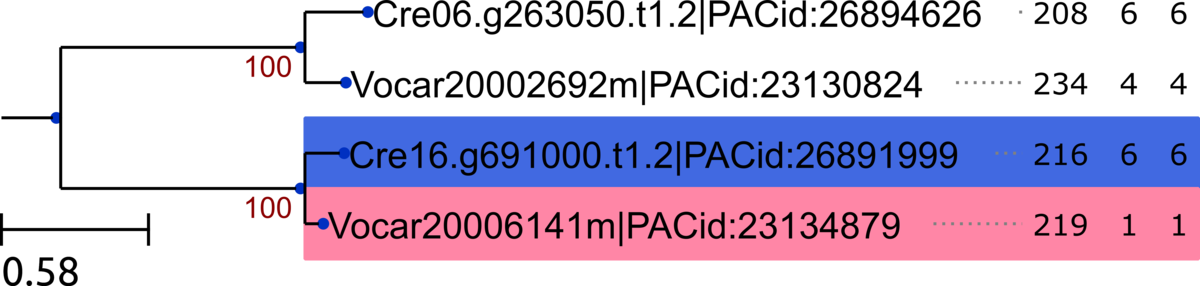
**

**group_41**

**
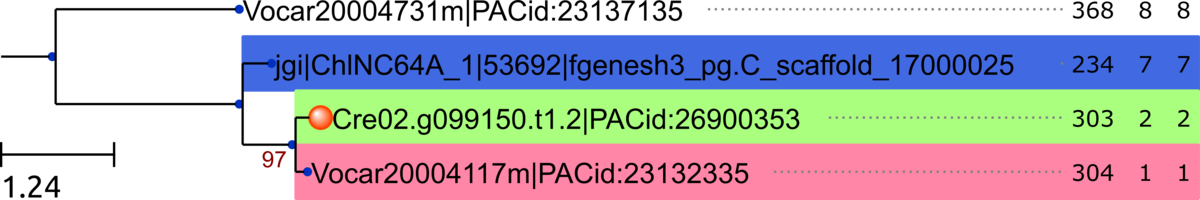
**

**group_42**

**
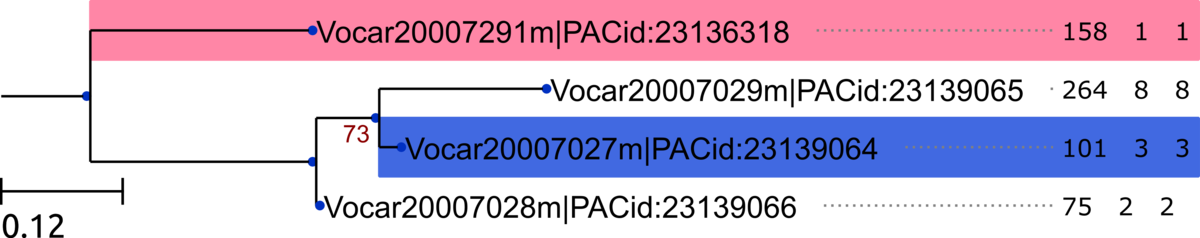
**

**group_43**

**
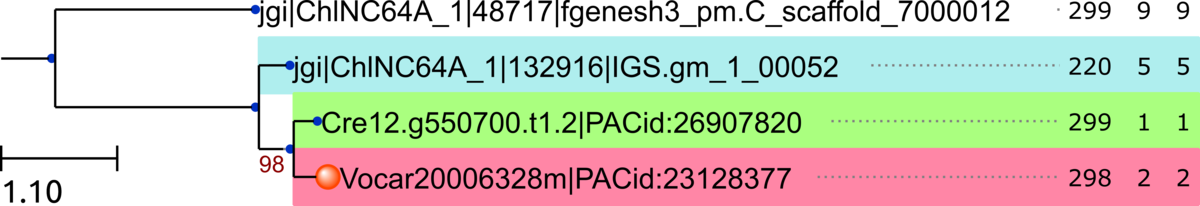
**

**group_44**

**
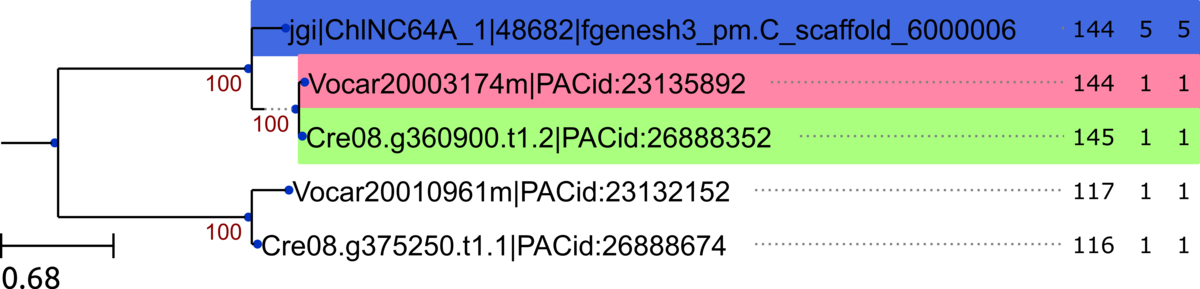
**

**group_45**

**
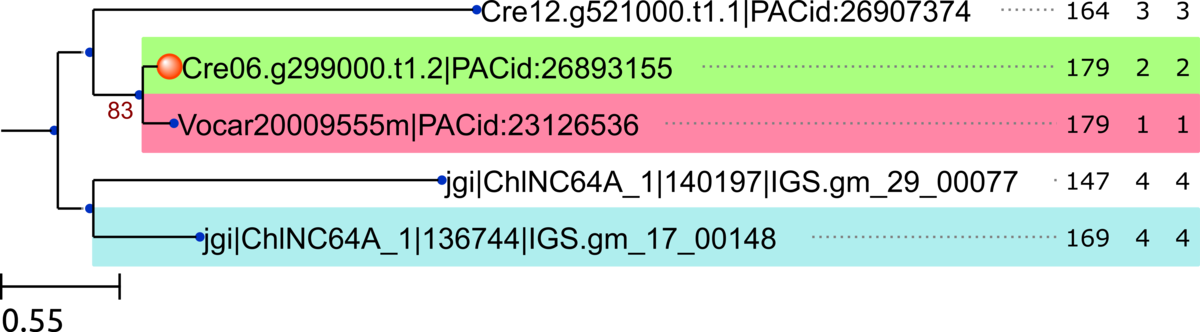
**

**group_46**

**
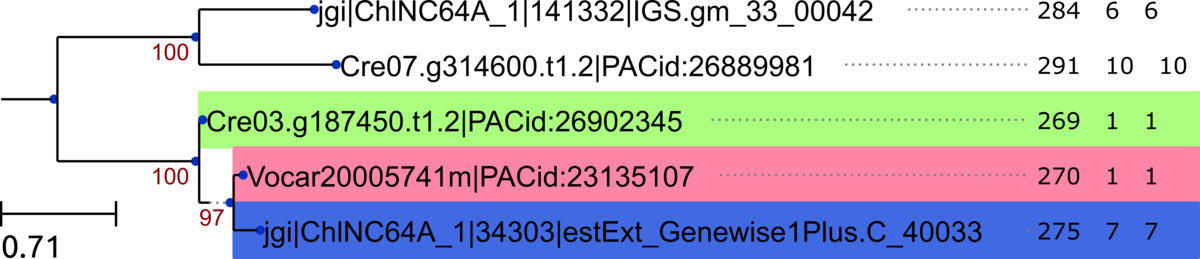
**

**group_47**

**
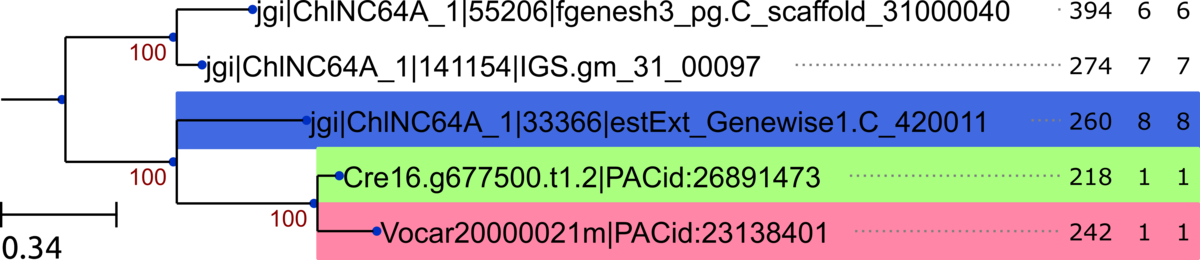
**

**group_48**

**
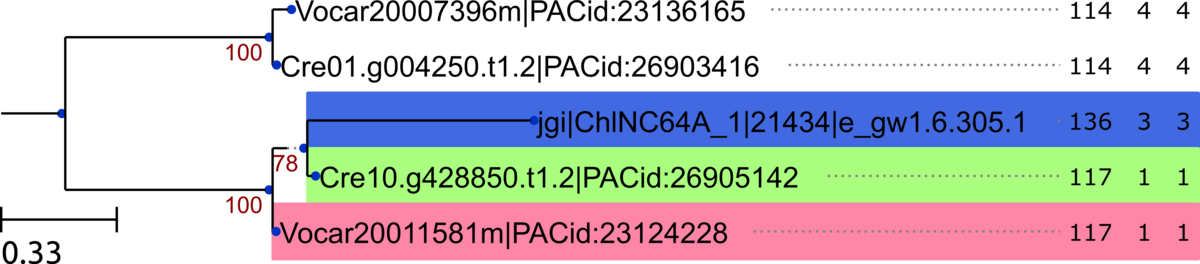
**

**group_49**

**
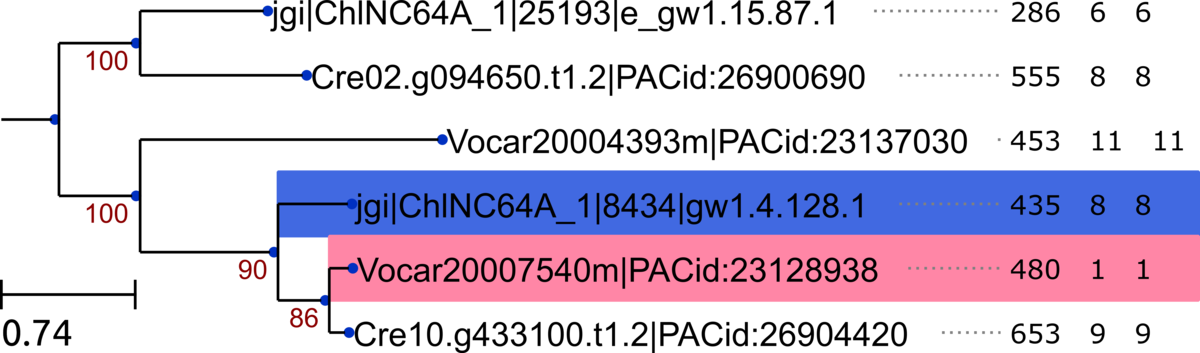
**

**group_50**

**
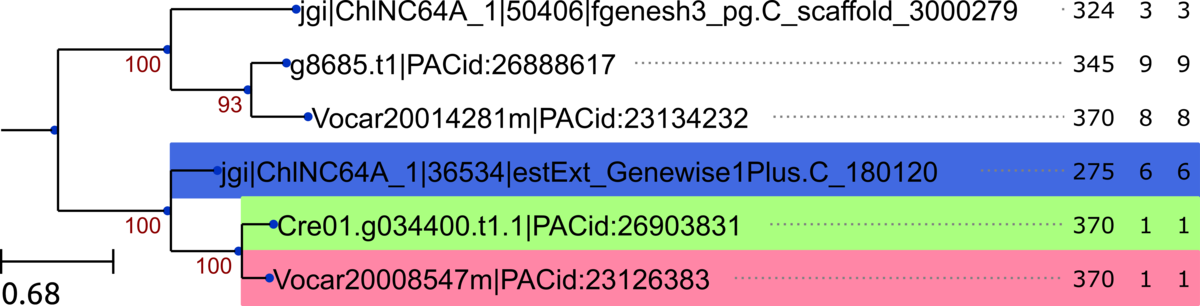
**

**group_51**

**
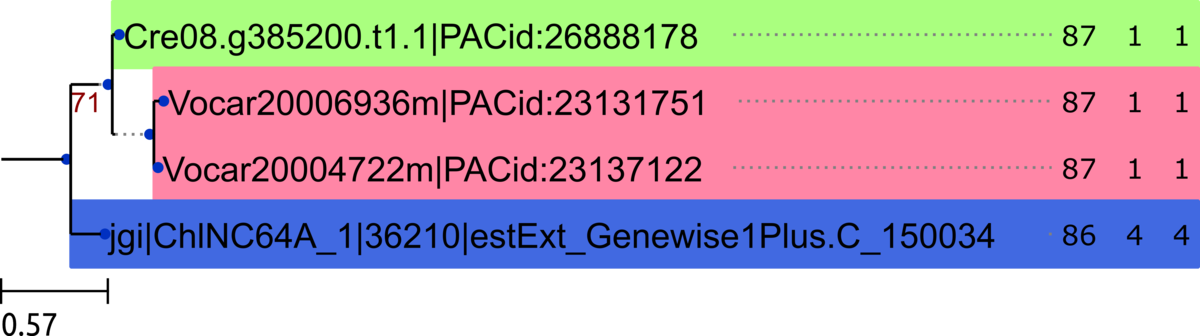
**

**group_53**

**
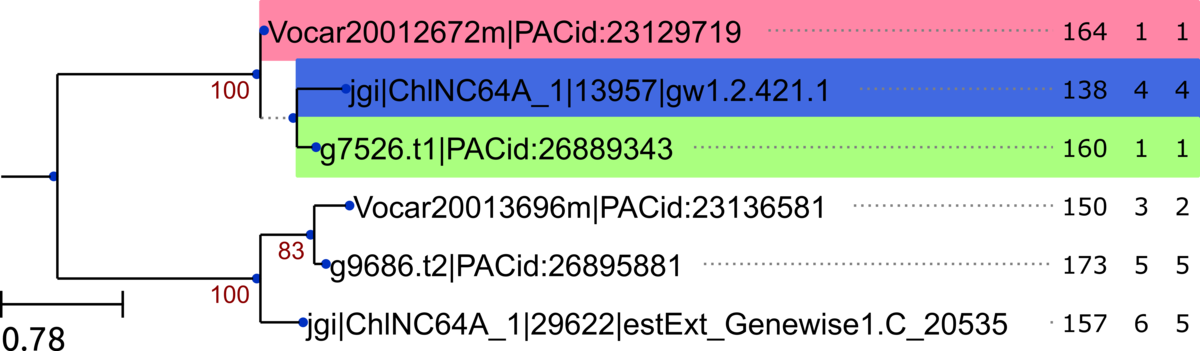
**

**group_54**

**
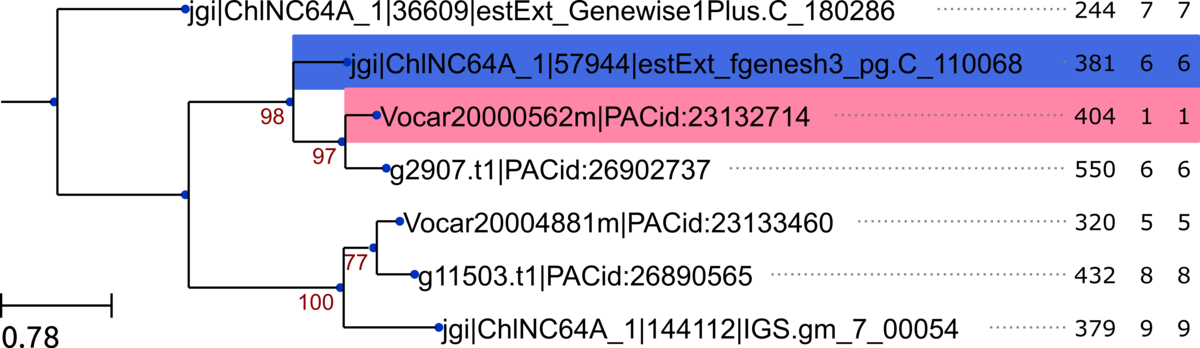
**

**group_55**

**
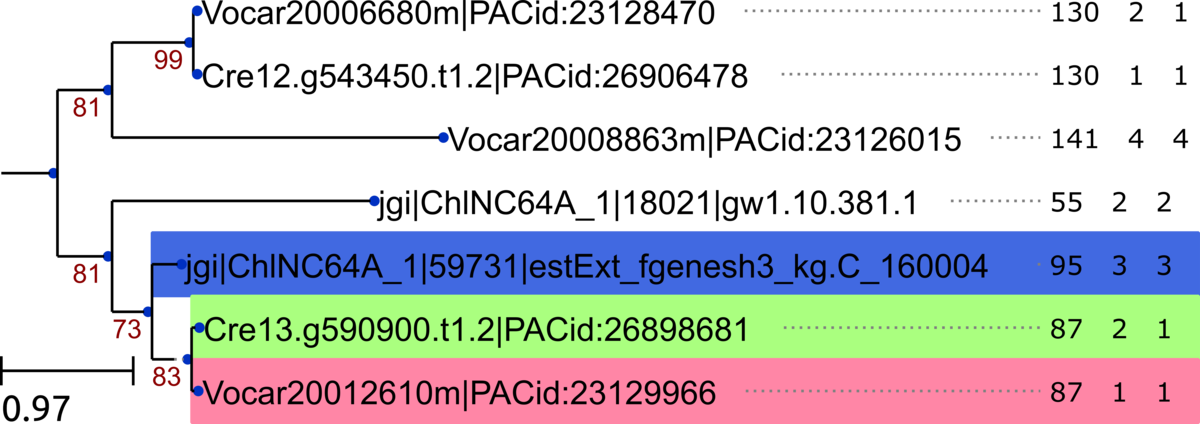
**

**group_56**

**
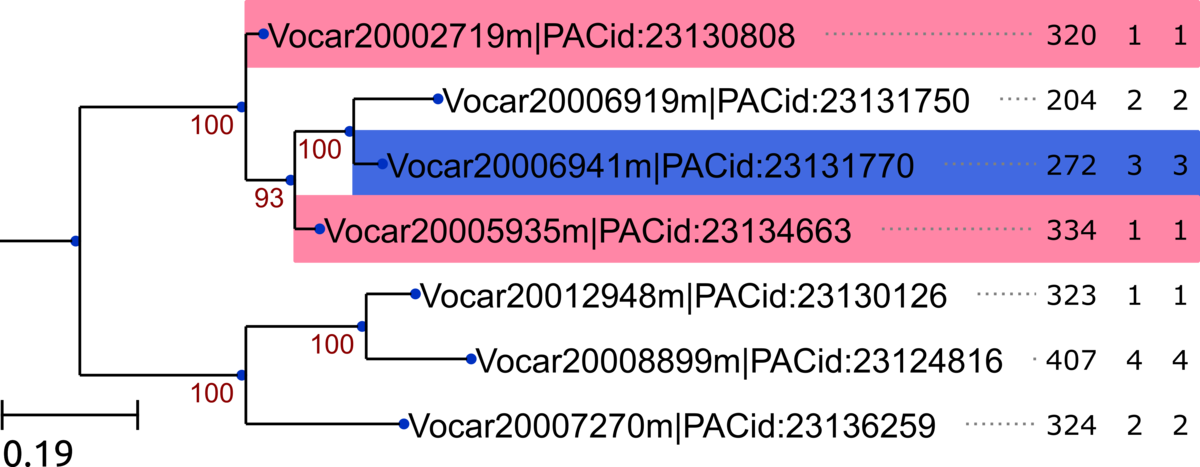
**

**group_57**

**
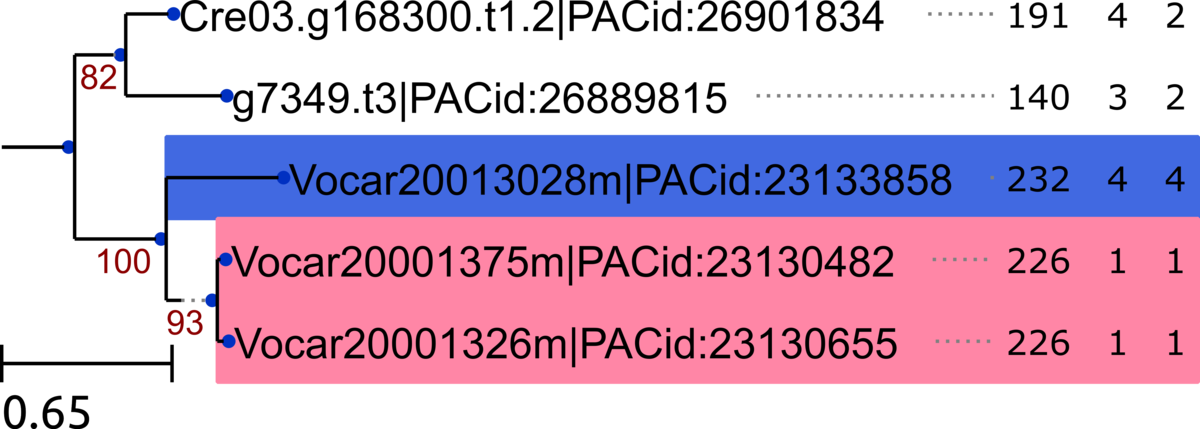
**

**group_58**

**
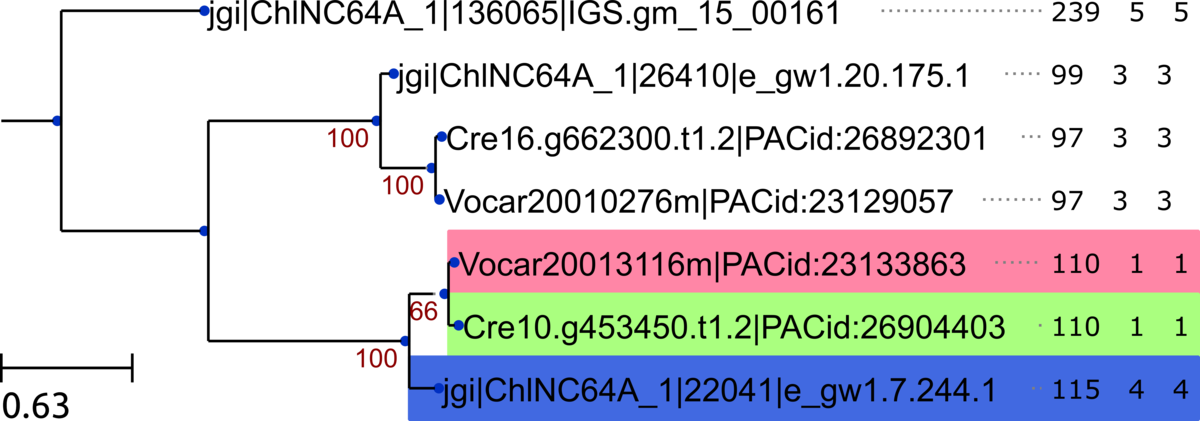
**

**group_59**

**
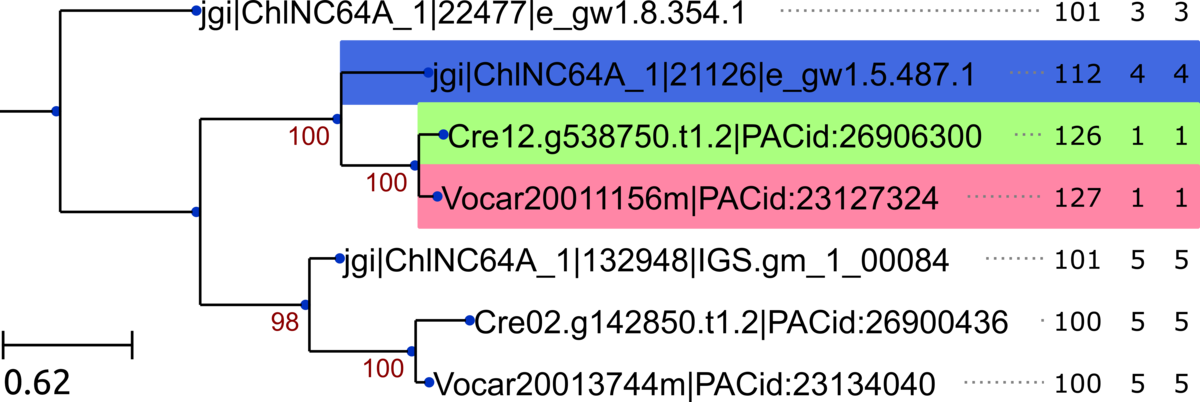
**

**group_60**

**
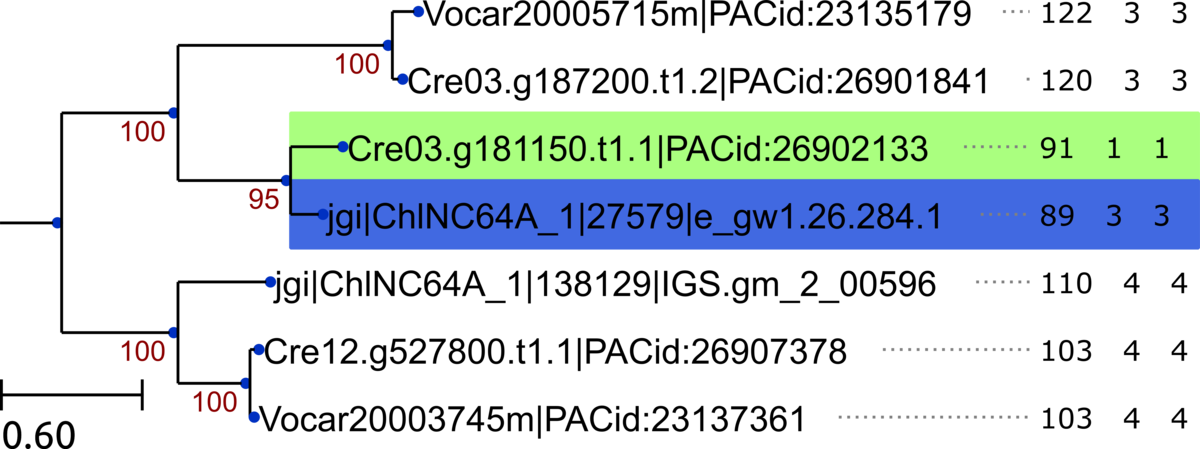
**

**group_61**

**
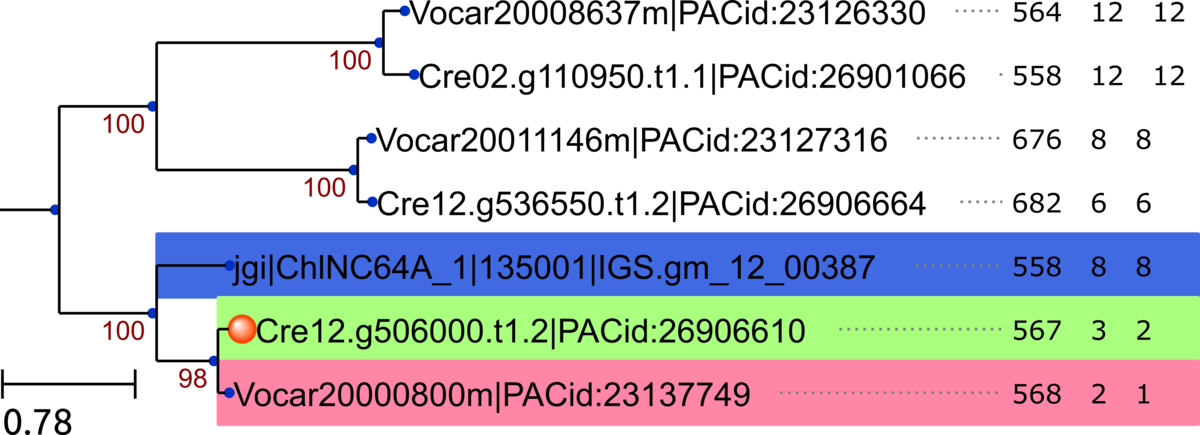
**

**group_62**

**
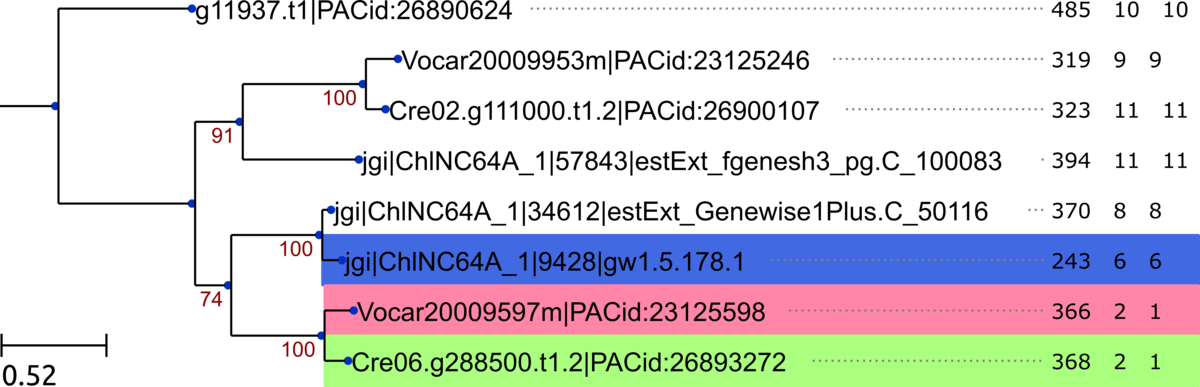
**

**group_63**

**
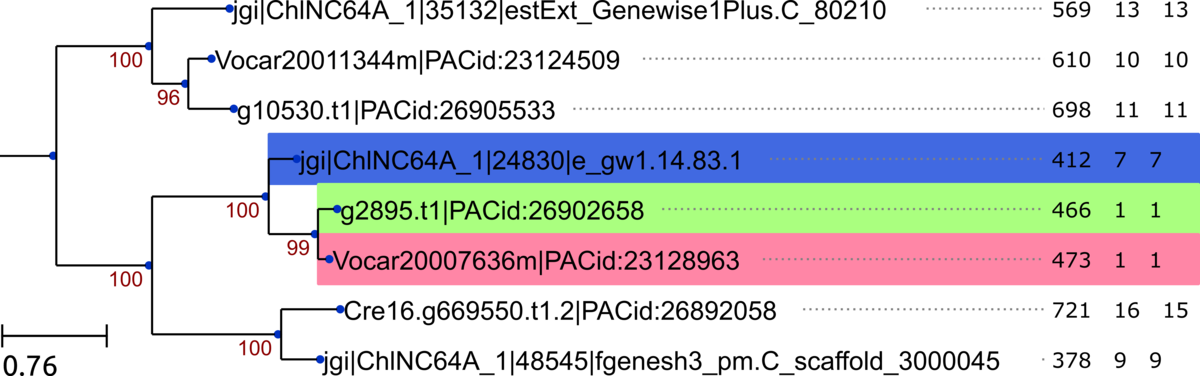
**

**group_64**

**
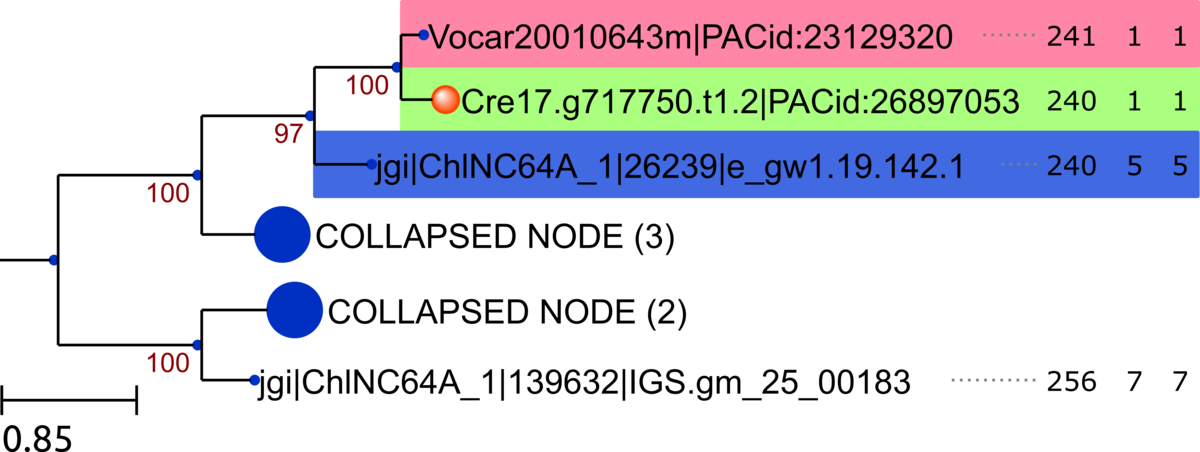
**

**group_65**

**
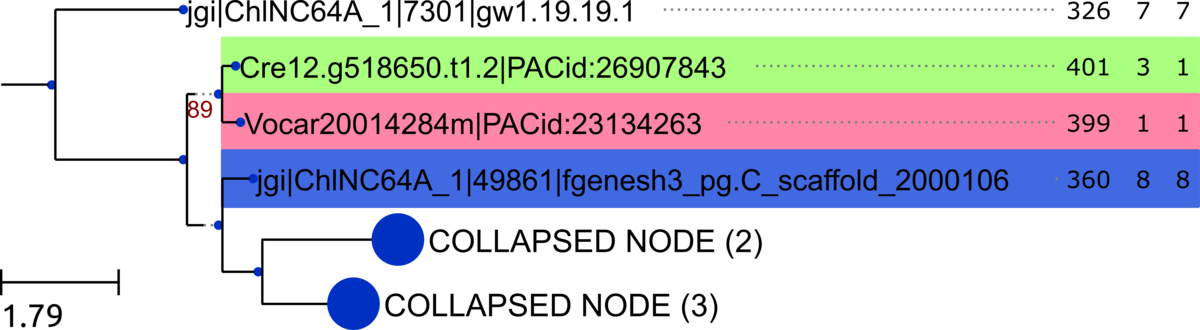
**

**group_66**

**
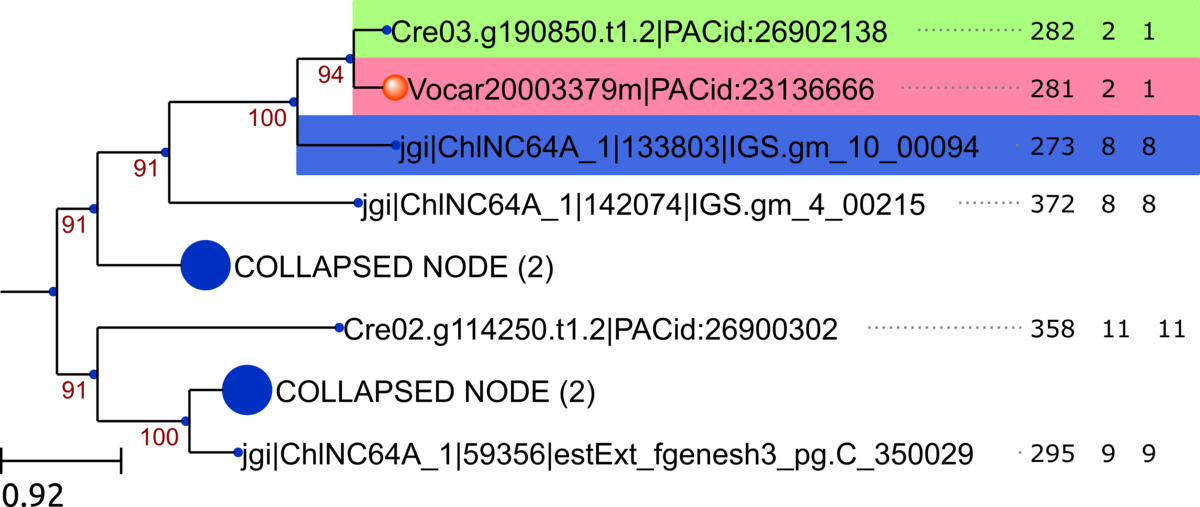
**

**group_67**

**
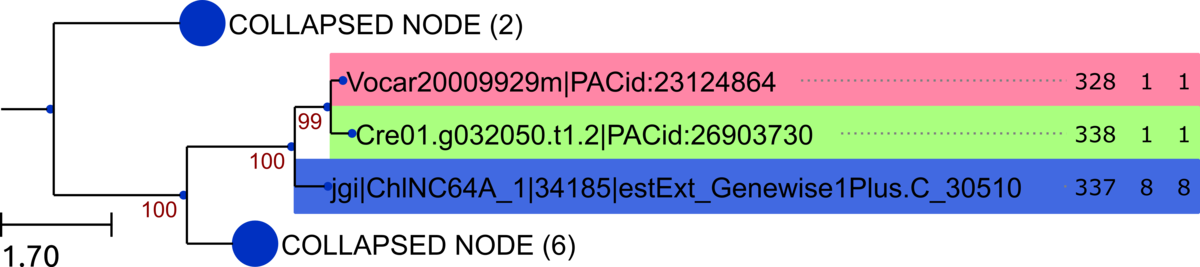
**

**group_68**

**
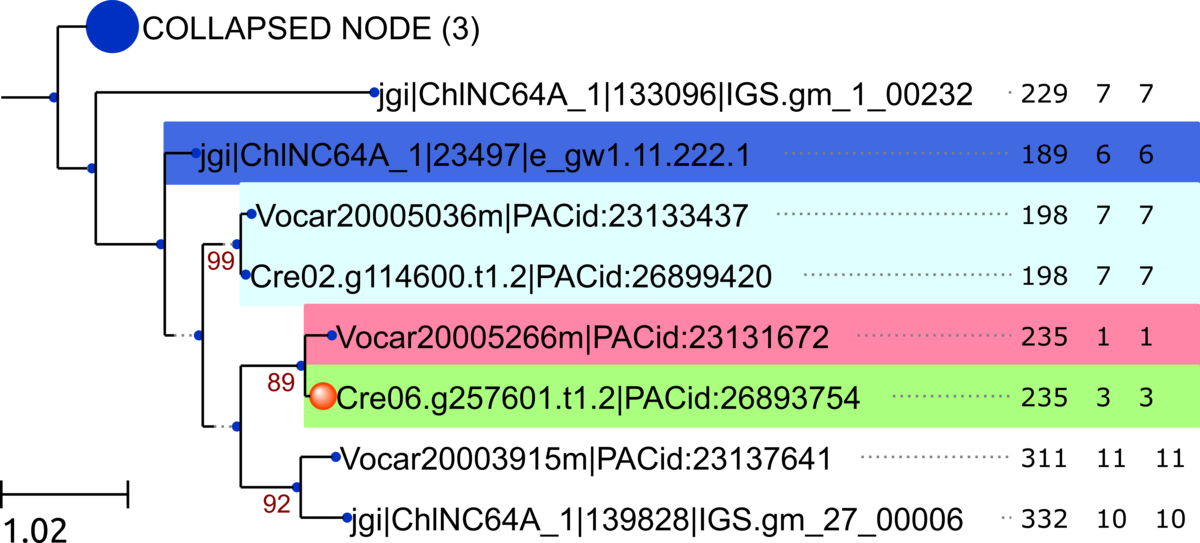
**

**group_69**

**
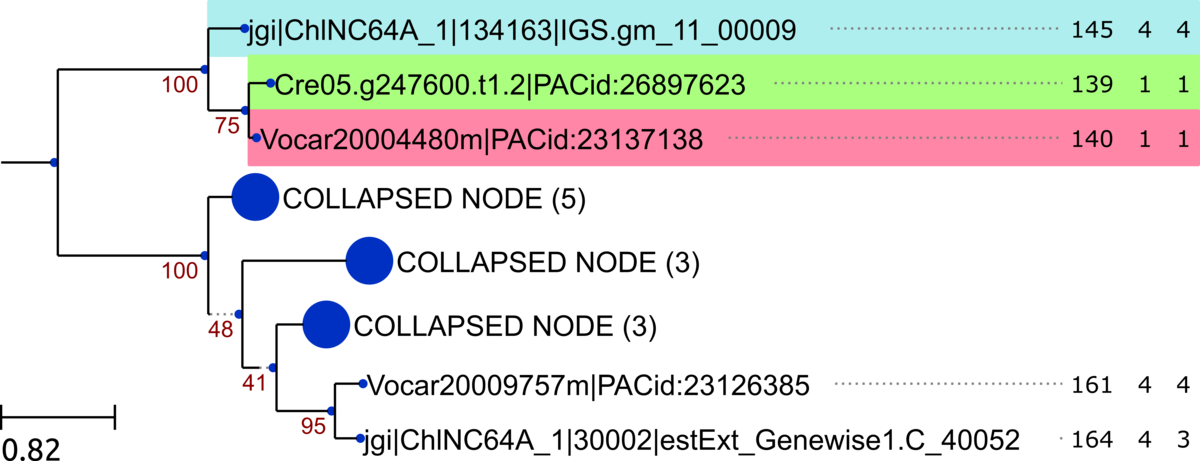
**

**group_71**

**
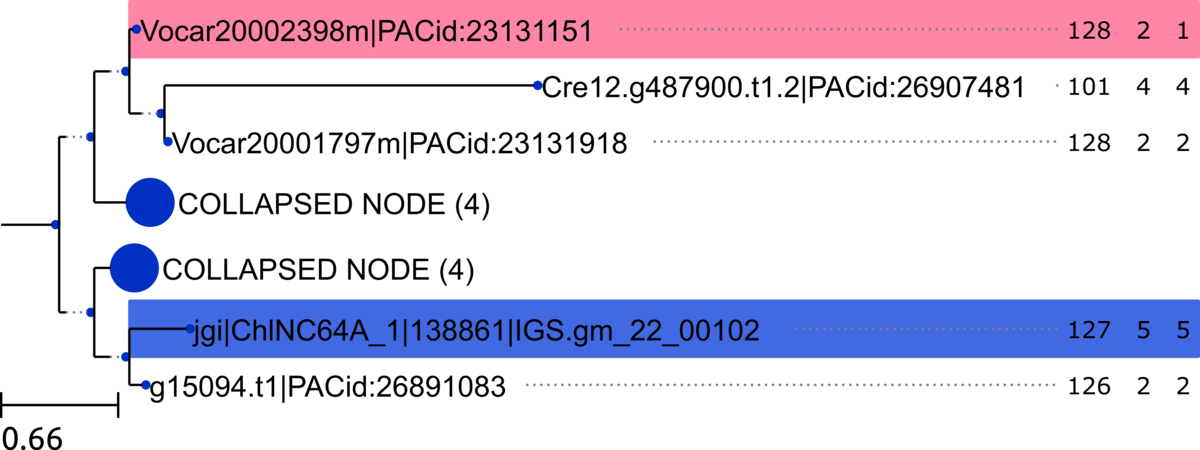
**

**group_72**

**
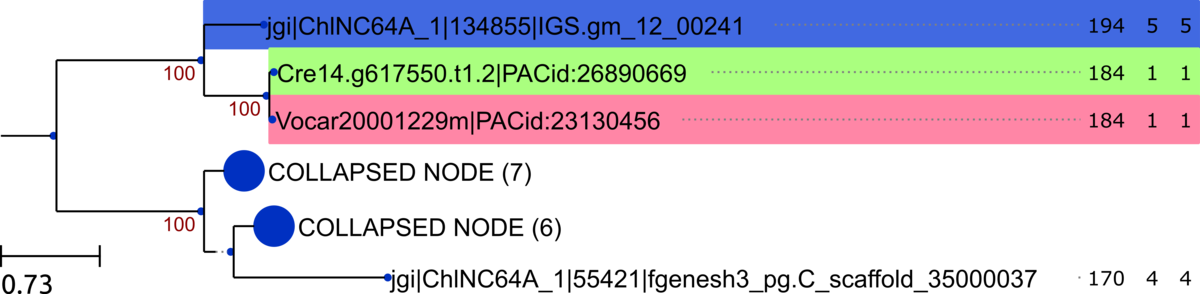
**

**group_73**

**
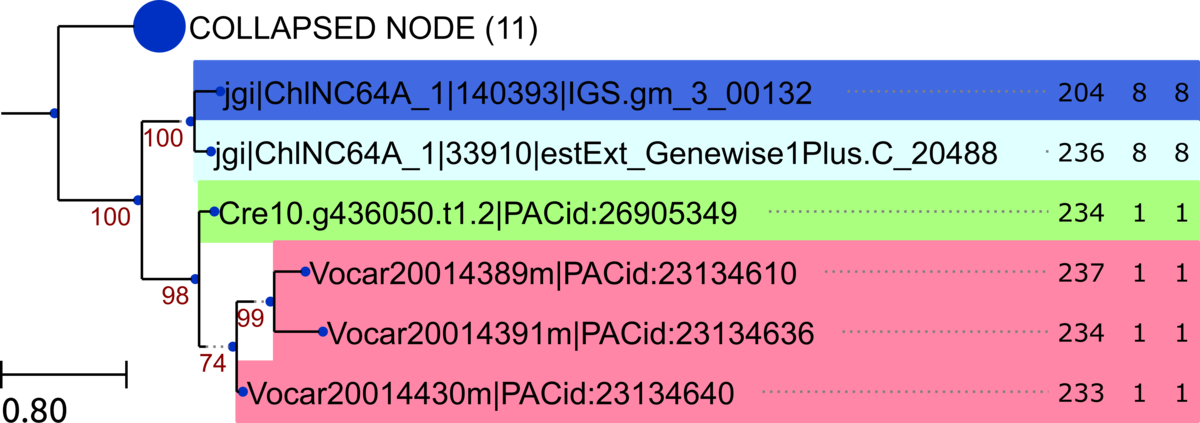
**

**group_74**

**
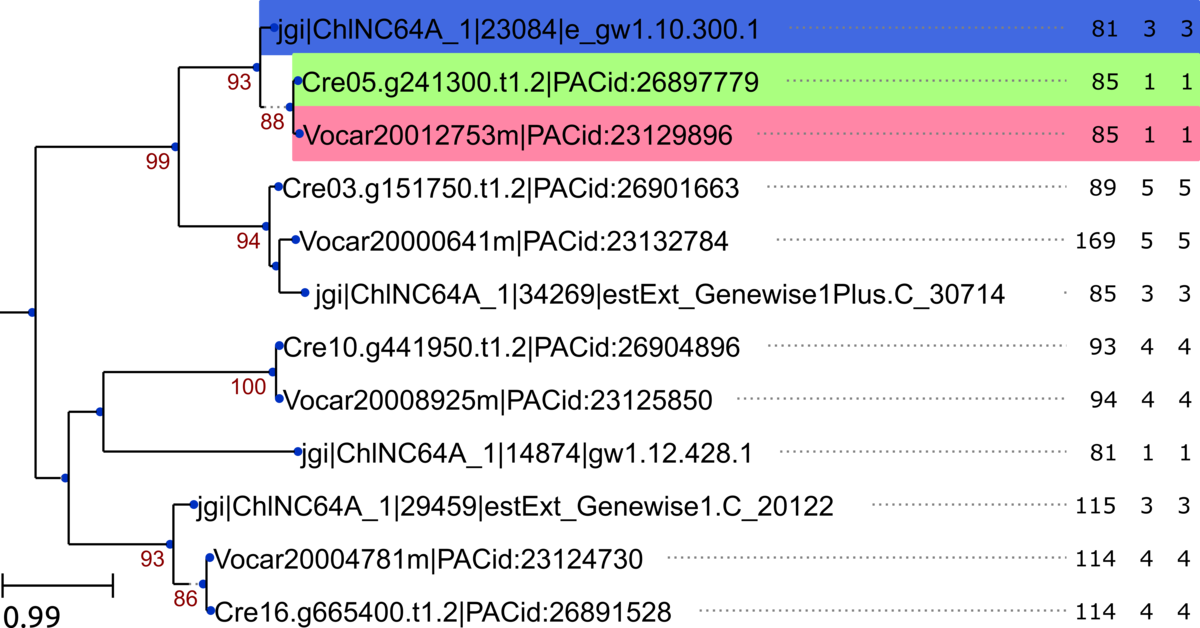
**

**group_77**

**
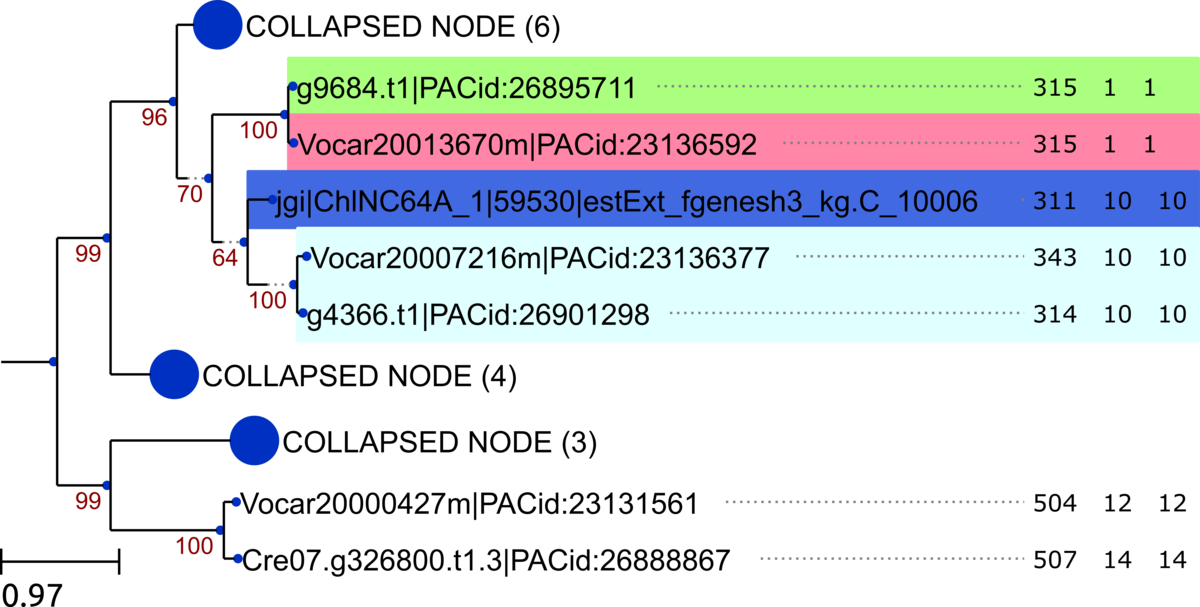
**

**group_78**

**
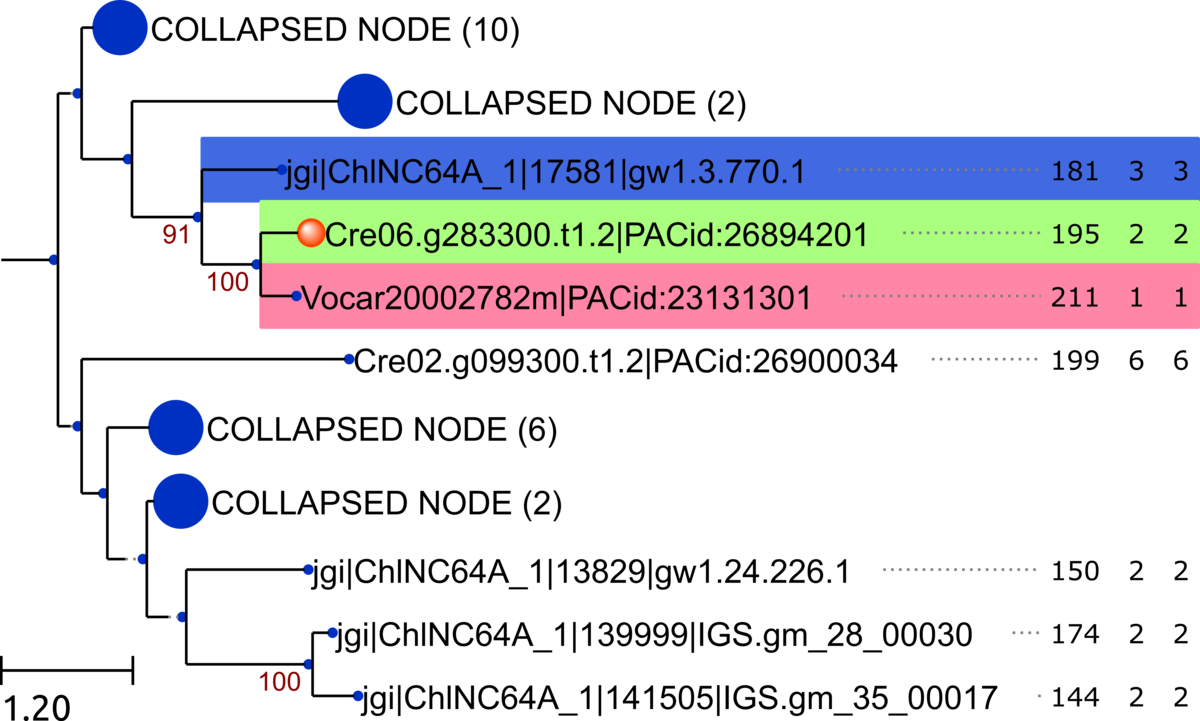
**

**group_79**

**
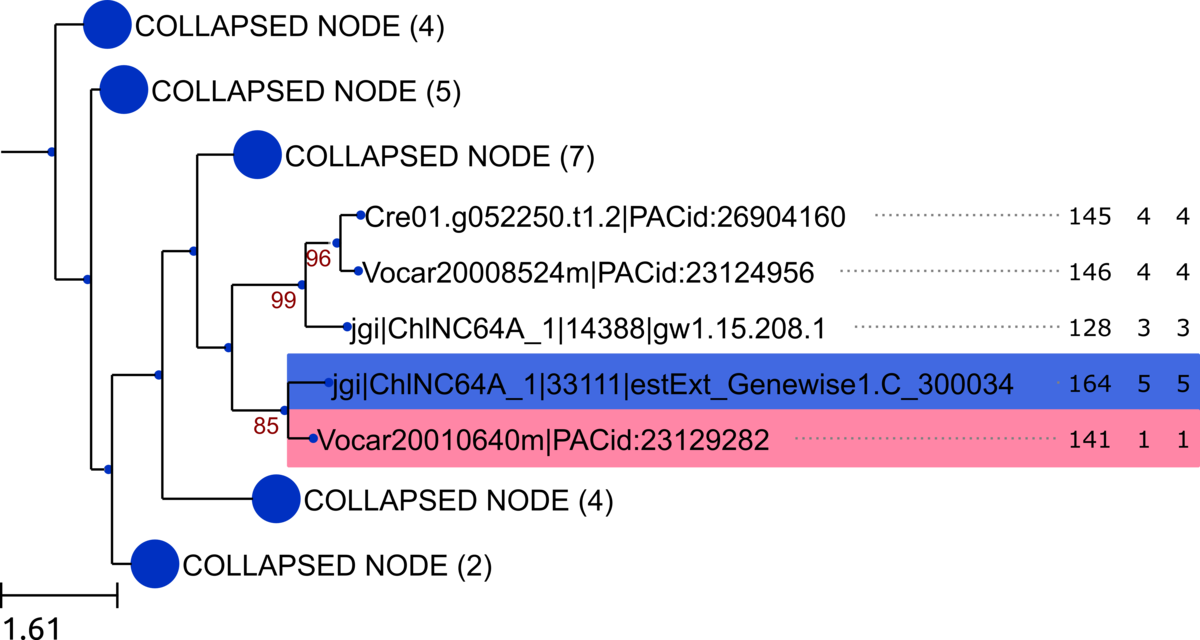
**

**group_80**

**
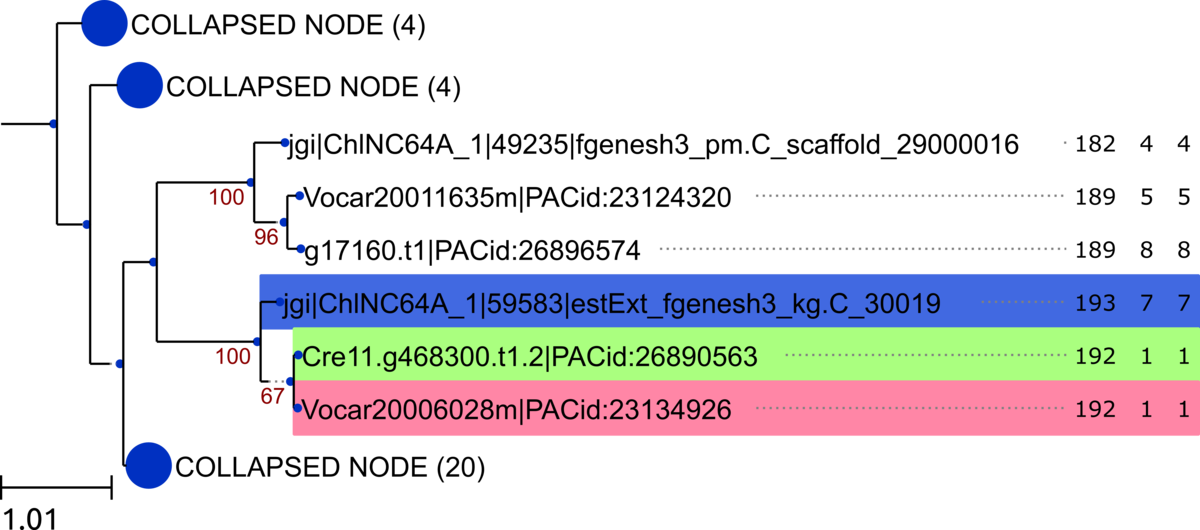
**

**group_81**

**
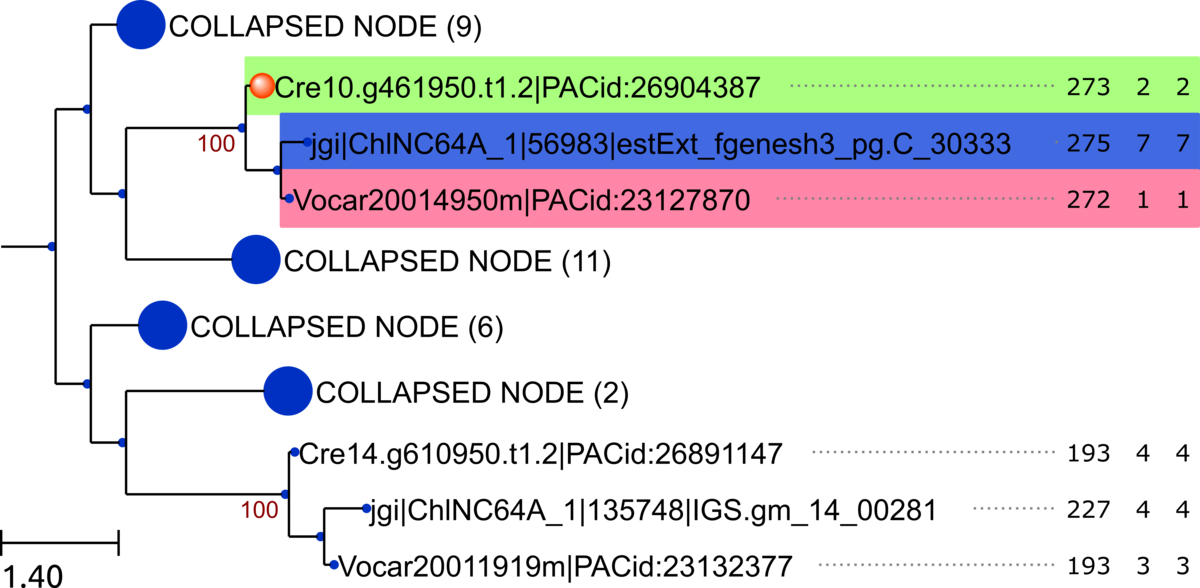
**

**group_82**

**
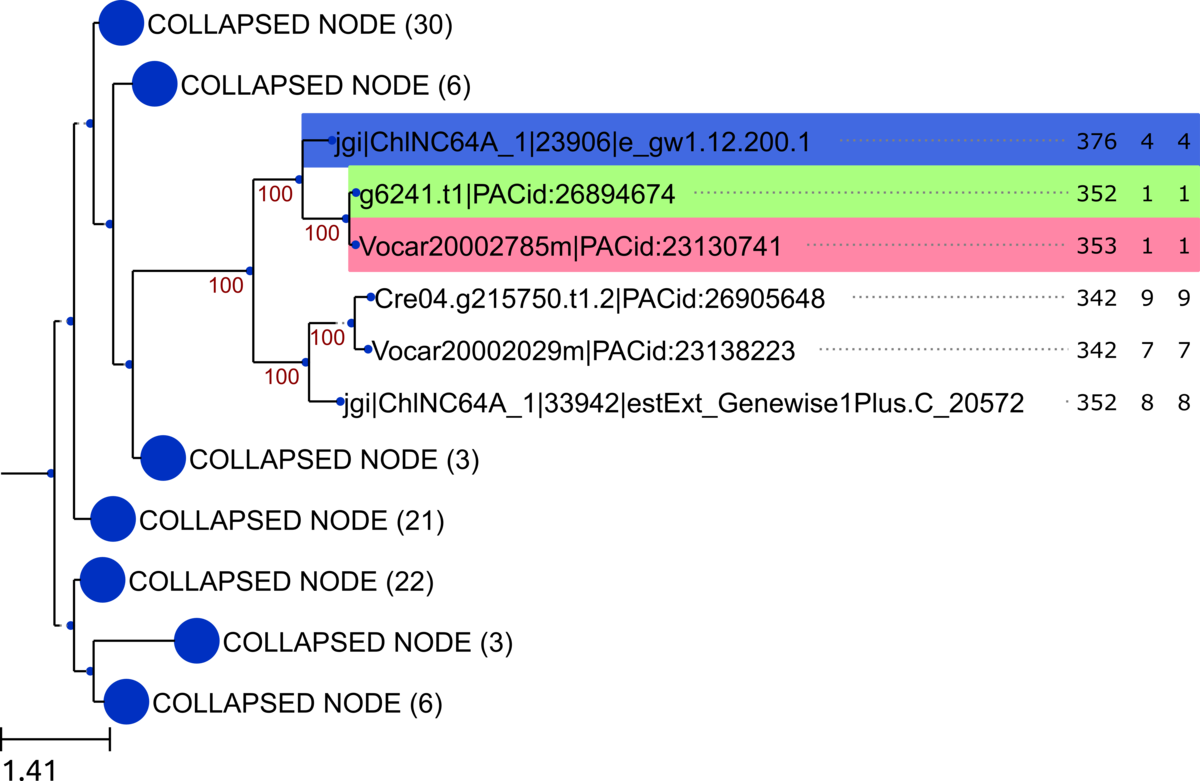
**

**Figure S2.** Maximum likelihood trees of the multi-species clusters containing retrogene candidates constructed with InParanoid. Phylogenetic relationships calculated with RAxML, based on alignments of amino acid sequences. Color code: light red – *Volvox* retrogene candidate, light green – *Chlamydomonas* retrogene candidate, blue – predicted parental gene. Orange circles indicate retrogene candidates identified manually, e.g. intronized, thus escaping the retrogene-finding strategy. Values in each OTU indicate length of encoded protein, number of exons, and number of coding exons. Support bootstrap values ≥ 50 for the calculated ML tree are shown on branches. The scale bars indicate the average number of amino acid substitutions per site. In case of large InParanoid-based homologous groups, distantly related clades were collapsed. Number of members of each collapsed clade is shown in brackets. All trees rooted with midpoint rooting approach.


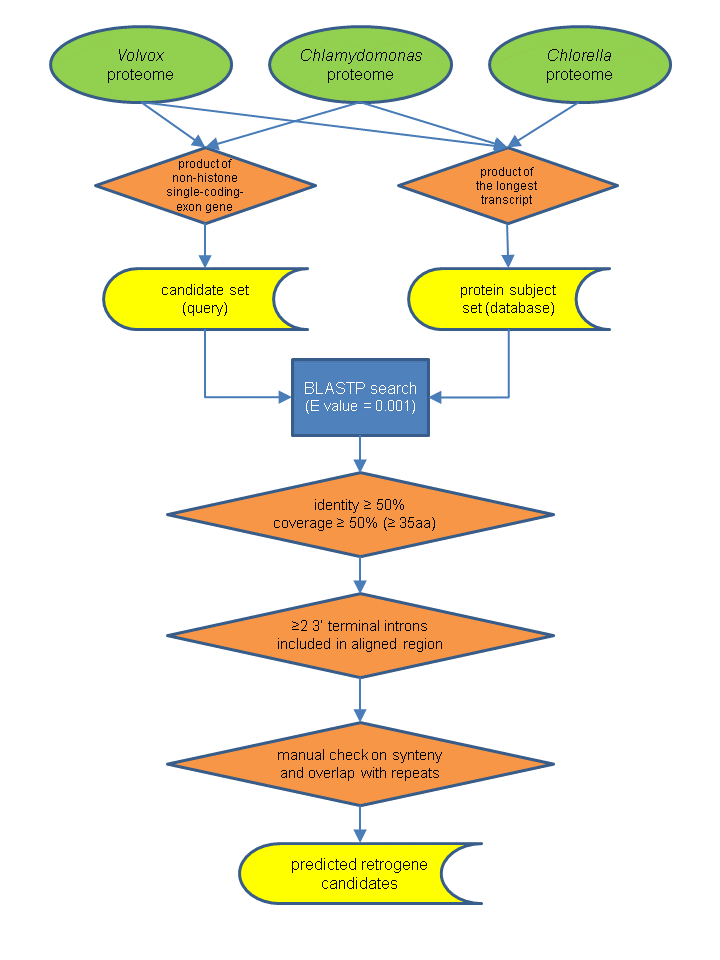


**Figure S3.** Identification of retrogene candidates in *Volvox* and *Chlamydomonas* genomes.

**Table S1.** Composition of multi-species homologous genes clusters containing the predicted retrogene candidates, constructed using InParanoid.

| Group | # of members | Retrogenes | Cre retrogenes | Vca retrogenes |
| --- | --- | --- | --- | --- |
| group_1 | 2 | Cre06.g296600 | 1 |  |
| group_2 | 2 | Vocar20002551m.g |  | 1 |
| group_3 | 2 | Vocar20003468m.g |  | 1 |
| group_4 | 2 | Vocar20004839m.g |  | 1 |
| group_5 | 2 | Vocar20000505m.g |  | 1 |
| group_6 | 2 | Cre12.g545650 | 1 |  |
| group_7 | 2 | Vocar20007587m.g |  | 1 |
| group_8 | 2 | Vocar20010753m.g |  | 1 |
| group_9 | 2 | Vocar20001040m.g |  | 1 |
| group_10 | 3 | Vocar20012302m.g | 1 | 1 |
| group_11 | 3 | Cre13.g585150, Vocar20009827m.g | 1 | 1 |
| group_12 | 3 | Vocar20011030m.g, Cre08.g382500 | 1 | 1 |
| group_13 | 3 | Cre01.g028150 | 1 | 1 |
| group_14 | 3 | Cre16.g692750 | 1 | 1 |
| group_15 | 3 | Vocar20014754m.g |  | 1 |
| group_16 | 3 | g8321, Vocar20012009m.g | 1 | 1 |
| group_17 | 3 | Vocar20006486m.g |  | 1 |
| group_18 | 3 | Vocar20003066m.g, Cre14.g620350 | 1 | 1 |
| group_19 | 3 | Vocar20011569m.g, g10494 | 1 | 1 |
| group_20 | 3 | g5356, Vocar20004414m.g | 1 | 1 |
| group_21 | 3 | Vocar20009258m.g | 1 | 1 |
| group_22 | 3 | Vocar20011462m.g, Cre17.g712400 | 1 | 1 |
| group_23 | 3 | Vocar20001853m.g | 1 | 1 |
| group_24 | 3 | Cre17.g735950 | 1 | 1 |
| group_25 | 3 | g5492, Vocar20000483m.g | 1 | 1 |
| group_26 | 3 | g15174 | 1 |  |
| group_27 | 3 | Vocar20002191m.g, g11264 | 1 | 1 |
| group_28 | 3 | Vocar20010236m.g | 1 | 1 |
| group_29 | 3 | Vocar20013082m.g, Cre02.g077300 | 1 | 1 |
| group_30 | 3 | Cre11.g481450 | 1 | 1 |
| group_31 | 3 | g15805.t1\|PACid:26892585 | 1 |  |
| group_32 | 3 | Vocar20009994m.g | 1 | 1 |
| group_33 | 3 | Vocar20003435m.g |  | 1 |
| group_34 | 3 | Vocar20013455m.g | 1 | 1 |
| group_35 | 3 | Vocar20011468m.g |  | 1 |
| group_36 | 3 | Vocar20004273m.g |  | 1 |
| group_37 | 4 | Vocar20008595m.g, Cre01.g029300 | 1 | 1 |
| group_38 | 4 | Vocar20013366m.g, Cre12.g494450 | 1 | 1 |
| group_39 | 4 | Vocar20012865m.g | 1 | 1 |
| group_40 | 4 | Vocar20006141m.g |  | 1 |
| group_41 | 4 | Vocar20004117m.g | 1 | 1 |
| group_42 | 4 | Vocar20007291m.g |  | 1 |
| group_43 | 4 | Cre12.g550700 | 1 | 1 |
| group_44 | 5 | Vocar20003174m.g, Cre08.g360900 | 1 | 1 |
| group_45 | 5 | Vocar20009555m.g | 1 | 1 |
| group_46 | 5 | Vocar20005741m.g, Cre03.g187450 | 1 | 1 |
| group_47 | 5 | Vocar20000021m.g, Cre16.g677500 | 1 | 1 |
| group_48 | 5 | Vocar20011581m.g, Cre10.g428850 | 1 | 1 |
| group_49 | 6 | Vocar20007540m.g |  | 1 |
| group_50 | 6 | Cre01.g034400, Vocar20008547m.g | 1 | 1 |
| group_51 | 4 | Vocar20004722m.g, Vocar20006936m.g, Cre08.g385200 | 1 | 2 |
| group_52 | 2 | Vocar20000705m.g |  | 1 |
| group_53 | 6 | Vocar20012672m.g, g7526 | 1 | 1 |
| group_54 | 7 | Vocar20000562m.g |  | 1 |
| group_55 | 7 | Cre13.g590900, Vocar20012610m.g | 1 | 1 |
| group_56 | 7 | Vocar20002719m.g, Vocar20005935m.g |  | 2 |
| group_57 | 5 | Vocar20001375m.g, Vocar20001326m.g |  | 2 |
| group_58 | 7 | Cre10.g453450, Vocar20013116m.g | 1 | 1 |
| group_59 | 7 | Vocar20011156m.g, Cre12.g538750 | 1 | 1 |
| group_60 | 7 | Cre03.g181150 | 1 |  |
| group_61 | 7 | Vocar20000800m.g | 1 | 1 |
| group_62 | 8 | Vocar20009597m.g, Cre06.g288500 | 1 | 1 |
| group_63 | 8 | Vocar20007636m.g, g2895 | 1 | 1 |
| group_64 | 9 | Vocar20010643m.g | 1 | 1 |
| group_65 | 9 | Vocar20014284m.g, Cre12.g518650 | 1 | 1 |
| group_66 | 10 | Cre03.g190850 | 1 | 1 |
| group_67 | 11 | Cre01.g032050, Vocar20009929m.g | 1 | 1 |
| group_68 | 11 | Vocar20005266m.g | 1 | 1 |
| group_69 | 16 | Cre05.g247600, Vocar20004480m.g | 1 | 1 |
| group_70 | 2 | Cre06.g306350 | 1 |  |
| group_71 | 14 | Vocar20002398m.g |  | 1 |
| group_72 | 17 | Vocar20001229m.g, Cre14.g617550 | 1 | 1 |
| group_73 | 17 | Vocar20014430m.g, Vocar20014389m.g, Vocar20014391m.g, Cre10.g436050 | 1 | 3 |
| group_74 | 18 | Vocar20012753m.g, Cre05.g241300 | 1 | 1 |
| group_75 | 3 | Vocar20008048m.g, Cre10.g455300 | 1 | 1 |
| group_76 | 3 | Vocar20002263m.g |  | 1 |
| group_77 | 20 | g9684, Vocar20013670m.g | 1 | 1 |
| group_78 | 27 | Vocar20002782m.g | 1 | 1 |
| group_79 | 27 | Vocar20010640m.g |  | 1 |
| group_80 | 34 | Cre11.g468300, Vocar20006028m.g | 1 | 1 |
| group_81 | 34 | Vocar20014950m.g | 1 | 1 |
| group_82 | 97 | Vocar20002785m.g, g6241 | 1 | 1 |
| **TOTAL** | 82 | 141 | 60 | 81 |

**Table S2.** Identified retrogene candidates and their homologs that are most likely retrogenes that underwent intron gain events.

| **Predicted retrogene candidate** | **Exons/CDSs** | **Parent** | **Exons/CDSs** | **Homologous retrogene candidate with intron gain** | **Exons/CDSs** | **Comment** |
| --- | --- | --- | --- | --- | --- | --- |
| Cre12.g550700 | 1/1 | jgi\|ChlNC64A_1\|132916\|IGS.gm_1_00052 | 5/5 | Vocar20006328m.g | 2/2 | Syntenic to its *Chlamydomonas* homolog |
| Vocar20000800m.g | 2/1 | jgi\|ChlNC64A_1\|135001\|IGS.gm_12_00387 | 8/8 | Cre12.g506000.t1.2 | 3/2 | Syntenic to its *Volvox* homolg |
| Vocar20009555m.g | 1/1 | jgi\|ChlNC64A_1\|136744\|IGS.gm_17_00148 | 4/4 | Cre06.g299000.t1.2 | 2/2 | Syntenic to its *Volvox* homolg |
| Vocar20013455m.g | 1/1 | jgi\|ChlNC64A_1\|17245\|gw1.14.448.1 | 4/4 | Cre06.g290000.t1.1 | 2/2 | Syntenic to its *Volvox* homolg |
| Vocar20002782m.g | 1/1 | jgi\|ChlNC64A_1\|17581\|gw1.3.770.1 | 3/3 | Cre06.g283300.t1.2 | 2.2 | Syntenic to its *Volvox* homolg |
| Vocar20001853m.g | 1/1 | jgi\|ChlNC64A_1\|19181\|e_gw1.2.429.1 | 3/3 | Cre05.g246800.t1.2 | 2/2 | No synteny (possible relocation) |
| Vocar20005266m.g | 1/1 | jgi\|ChlNC64A_1\|23497\|e_gw1.11.222.1 | 6/6 | Cre06.g257601.t1.2 | 3/3 | No synteny (possible relocation) |
| Vocar20012865m.g | 1/1 | jgi\|ChlNC64A_1\|24122\|e_gw1.12.252.1 | 5/5 | Cre17.g732850.t1.2 | 3.3 | Syntenic to its *Volvox* homolg |
| Vocar20009258m.g | 1/1 | jgi\|ChlNC64A_1\|32279\|estExt_Genewise1.C_180247 | 3/3 | Cre01.g040450.t1.2 | 2/2 | No synteny (possible relocation) |
| Vocar20009994m.g | 1/1 | jgi\|ChlNC64A_1\|32442\|estExt_Genewise1.C_210068 | 4/4 | Cre06.g288750.t1.2 | 2/2 | Syntenic to its *Volvox* homolg |
| Vocar20012302m.g | 1/1 | jgi\|ChlNC64A_1\|48524\|fgenesh3_pm.C_scaffold_3000024 | 6/6 | Cre16.g692050.t1.1 | 2/2 | No synteny (possible relocation) |
| Vocar20010236m.g | 1/1 | jgi\|ChlNC64A_1\|49159\|fgenesh3_pm.C_scaffold_26000005 | 8/8 | Cre16.g663100.t1.1 | 2/2 | No synteny (possible relocation) |
| Vocar20004117m.g | 1/1 | jgi\|ChlNC64A_1\|53692\|fgenesh3_pg.C_scaffold_17000025 | 7/7 | Cre02.g099150.t1.2 | 2/2 | No synteny (possible relocation) |
| Vocar20014950m.g | 1/1 | jgi\|ChlNC64A_1\|56983\|estExt_fgenesh3_pg.C_30333 | 7/7 | Cre10.g461950.t1.2 | 2/2 | Syntenic to its *Volvox* homolg |
